# Metabolome and transcriptome-wide effects of the carbon storage regulator A in enteropathogenic *Escherichia coli*

**DOI:** 10.1038/s41598-018-36932-w

**Published:** 2019-01-15

**Authors:** Volker Berndt, Michael Beckstette, Marcel Volk, Petra Dersch, Mark Brönstrup

**Affiliations:** 1 Department of Chemical Biology, Helmholtz Centre for Infection Research and German Center for Infection Research (DZIF), Braunschweig, Germany; 2 Department of Molecular Infection Biology, Helmholtz Centre for Infection Research and German Center for Infection Research (DZIF), Braunschweig, Germany; 3 Biomolecular Drug Research Centre (BMWZ), Schneiderberg 38, 30167 Hannover, Germany

## Abstract

The carbon storage regulator A (CsrA) is a conserved global regulatory system known to control central carbon pathways, biofilm formation, motility, and pathogenicity. The aim of this study was to characterize changes in major metabolic pathways induced by CsrA in human enteropathogenic *Escherichia coli* (EPEC) grown under virulence factor-inducing conditions. For this purpose, the metabolomes and transcriptomes of EPEC and an isogenic *∆csrA* mutant derivative were analyzed by untargeted mass spectrometry and RNA sequencing, respectively. Of the 159 metabolites identified from untargeted GC/MS and LC/MS data, 97 were significantly (fold change ≥ 1.5; corrected p-value ≤ 0.05) regulated between the knockout and the wildtype strain. A lack of *csrA* led to an accumulation of fructose-6-phosphate (F6P) and glycogen synthesis pathway products, whereas metabolites in lower glycolysis and the citric acid cycle were downregulated. Associated pathways from the citric acid cycle like aromatic amino acid and siderophore biosynthesis were also negatively influenced. The nucleoside salvage pathways were featured by an accumulation of nucleosides and nucleobases, and a downregulation of nucleotides. In addition, a pronounced downregulation of lyso-lipid metabolites was observed. A drastic change in the morphology in the form of vesicle-like structures of the *∆csrA* knockout strain was visible by electron microscopy. Colanic acid synthesis genes were strongly (up to 50 fold) upregulated, and the abundance of colanic acid was 3 fold increased according to a colorimetric assay. The findings expand the scope of pathways affected by the *csrA* regulon and emphasize its importance as a global regulator.

## Introduction

Enteropathogenic *Escherichia coli* (EPEC) is a Gram-negative, foodborne human pathogen, which causes diarrhea in humans and is the major agent for infantile diarrhea worldwide. EPEC is responsible for up to two million deaths of children younger than 5 years in developing countries per year. EPEC forms micro-colonies in the small intestine and attaches to the epithelial cells, causing lesions and effacement of the underlying microvilli^1,2^. Effectors injected by a type III secretion system (T3SS) induce a reorganization of the host cytoskeleton to pedestal-like structures that allow the bacteria to persist on the cell surface^3,4^. EPEC is a close relative to enterohemorrhagic *E. coli* (EHEC), but does not produce Shiga toxins^5^.

Evolution developed various regulatory mechanisms that fine-tune catabolic and anabolic pathways in living cells in order to optimize the use of energy from external nutrition in response to changing conditions. Especially enteropathogens have to face competition with commensal bacteria and high and massive fluctuations of the nutritional composition. It has been shown that colonization of enteropathogenic *E. coli* is tightly linked to the utilization of simple sugars and small organic acids from degraded mucus or dietary fibers^6^. Consequently, it is important that they rapidly adjust their carbon metabolism to successfully establish the infection.

The impact of post-transcriptional regulatory systems on metabolic functions of enteropathogenic *E. coli*, as such exerted by the carbon storage regulator (Csr), is not well characterized. However, several studies on laboratory *E. coli* K-12 strains were published recently^7–12^. The Csr system consists of the dimeric mRNA binding protein CsrA and small regulatory RNAs, which inhibit CsrA activity. The regulatory function of CsrA is based on the binding of the protein to GGA motifs of the 5′- UTR of target mRNAs. The conserved region which shows a high affinity for CsrA contains a consensus sequence: RUA CAR GGA UGU^13^, whereof the ACA and GGA (underlined) motif is completely conserved in high affinity transcripts that were identified by SELEX, but is not completely conserved in native mRNAs or sRNAs. The majority of characterized GGA motifs overlap and/or are located near the ribosomal binding site of the target gene^14^. In many cases, CsrA recognizes two adjacent hairpin structures, which include GGA motifs residing in the single-stranded loop regions. Most frequently, one GGA motif is part of the ribosome binding site of the target gene, and CsrA-binding prevents the attachment of ribosomes, resulting in the downregulation of protein translation and a faster mRNA degradation^15^. On the other hand, CsrA can also act as a positive regulator of protein translation by stabilizing secondary structures of mRNA^15,16^. It is also known that CsrA enhances gene expression by protecting mRNA from RNAse E-mediated cleavage, as demonstrated e.g. for *flhDC* mRNA^17^. CsrA is a highly conserved regulatory protein and fulfills many regulatory functions especially in *Enterobacteriaceae*. For *E. coli* K-12 high throughput transcriptomics analysis were performed to assess the function of the Csr system^8,10,18^. Potts *et al*. could detect 457 direct CsrA targets by cross-linking immunoprecipitation-high-throughput sequencing (CLIP-Seq) which underline the global regulatory function of the Csr system. Among the them are well-known targets of the Csr system, but also novel CsrA targets including transcripts of the fatty acid biosynthesis pathway, various nutrition uptake systems, maintenance of the cell envelope integrity and regulation of iron uptake and metabolism were discovered. In addition, CsrA influence on the expression of 87 transcriptional regulators and 11 sensor kinases was detected, supporting a global effect on the transcriptional landscape of *E. coli*^8^. Sowa *et al*. used an Integrative FourD omics approach and could identify 136 potential direct CsrA targets, of which 50 were not detected previously^18^ (Dataset S2).

CsrA also plays a key role in growth-dependent metabolic pathways, motility and also in virulence in the enteropathogenic proteobacteria *Yersinia pseudotuberculosis* and *Salmonella typhimurium*^15^. For instance, it could be shown that *Y. pseudotuberculosis ∆csrA* deletion mutants are strongly inhibited in growth as well as in virulence, indicating that CsrA is a crucial factor in the regulation of bacterial fitness and host-bacterial interactions^19–21^. The regulatory function of CsrA concerning virulence regulation was also shown among *Salmonella* species for the regulation of the two distinct type III secretion systems (T3SSs) as well as for other *Enterobacteriaceae* and *Pseudomonads*. Therefore, efforts to explore CsrA as a drug target to treat bacterial infections have been initiated^22^.

CsrA also regulates virulence in EPEC by binding to the LEE4 mRNA leader, and by the indirect positive regulation of *escD*^23,24^. Both LEE4 and *escD* encode for translocators of the EPEC T3SS. Additionally CsrA is antagonized by the EPEC chaperone CesT, which is part of the LEE-pathogenicity island^25^. Also effects on other regulators and proteins with functions in virulence gene expression in EPEC were reported. Most knowledge of the EPEC CsrA regulon was gained from transcriptome data or mRNA binding assays, but did not capture effects on the bacterial metabolome^11,23^. The ability of CsrA to control the central carbon metabolism has been demonstrated by the analysis of metabolic fluxes in the nonpathogenic *E. coli* strains K12-MG1655, Nissle 1917, and in other *Enterobacteriaceae*^9,12,26,27^. However, a global overview about the CsrA-promoted coregulation of virulence factors and of metabolic pathways in pathogenic *E. coli* is still missing. To fill this gap, a combined analysis of the metabolome and the transcriptome sampled from the EPEC strain E2348/69 and its *∆csrA* deficient derivate was performed in this study. The growth media were carefully selected to assure virulence gene expression of EPEC under study conditions. In order to obtain a wide coverage of metabolites in an unbiased manner, a combination of untargeted liquid chromatography-coupled mass spectrometry (LC-MS) in positive and negative ionization modes with gas chromatography-coupled mass spectrometry was applied, followed by matching of detected components to internal and external metabolite databases^28–30^. As shown below, the combined analysis of metabolome and transcriptome changes induced by the loss of *csrA* provides detailed insights into the influence of CsrA on central metabolic pathways and virulence-associated mechanisms such as regulation of T3SS and colanic acid synthesis.

## Results

To gain a global view of the influence of CsrA on the bacterial metabolism and co-regulated virulence gene expression, we investigated the metabolome as well as the transcript levels in the EPEC wildtype E2348/69 and its isogenic *∆csrA* mutant. In order to probe whether loss of *csrA* had an influence on the replication rate of EPEC, bacterial growth was monitored over the differential growth phases. For normalization of the meta-bolome experiments, the dry mass was correlated to the optical density (Table 1). Doubling time and growth rate in MOPS and M9 medium were strongly reduced for the *∆csrA* strain supplemented with glucose and casaminoacids (Fig. S1). The *∆csr51* strain of MG1655 showed a reduction in growth in M9 media with a maximal growth rate of 0,31 ± 0,01 h^−1^ ^10^, very similar to what has been observed for the EPEC 2348/69 Δ*csrA* strain with 0,43 ± 0,054 h^−1^. The growth curves in M9 and MOPS medium are shown in Fig. S1. The dry weight of the EPEC wildtype was comparable to previous studies with *E. coli* grown in LB medium (300 mg/l at an OD_600_ of 1)^31^, whereas the dry weight of the *ΔcsrA* strain was significantly reduced (Table 1); a similar growth phenotype was also observed in *E. coli* K-12 *csrA* mutants^10,32^. To assess the genetic stability of the *∆csrA* mutant, the mutant strain was grown in Kornberg medium, and colonies were stained for intracellular glycogen with iodine vapour or Lugol’s solution. The *∆csrA* mutant was slightly darker than the EPEC wildtype due to an increased accumulation of glycogen. Iodine stained colonies of the *∆csrA* mutant were homogenous, which implies that the EPEC Δ*csrA* mutant is not obviously genetically instable.

**Table 1:** Growth and OD_600_ to biomass correlation of EPEC E2348/69 wildtype and ΔcsrA strains in different growth media.

Strain	Medium	µ [h^−1^]	t_d_ [min]	Drymass [mg]*
*E2348/69 wt*	MOPS 0.05% CA	1.26 ± 0.036	32.3 ± 0.8	294.24 ± 23.38
*E2348/69 wt*	M9^#^ 0.05% CA	0.66 ± 0.042	64.7 ± 3.1	305.34 ± 29.44
*E2348/69 ΔcsrA*	MOPS 0.05% CA	0.66 ± 0.024	65.7 ± 2.7	263.76 ± 0.01
*E2348/69 ΔcsrA*	M9 0.05% CA	0.42 ± 0.054	95.1 ± 5.2	258.59 ± 7.38

µ: growth rate; t_d_: doubling time; CA: casamino acids; ^#^standard M9 medium composition *drymass in mg for 1 liter of culture at an OD_600_ of 1

**Figure 1:**
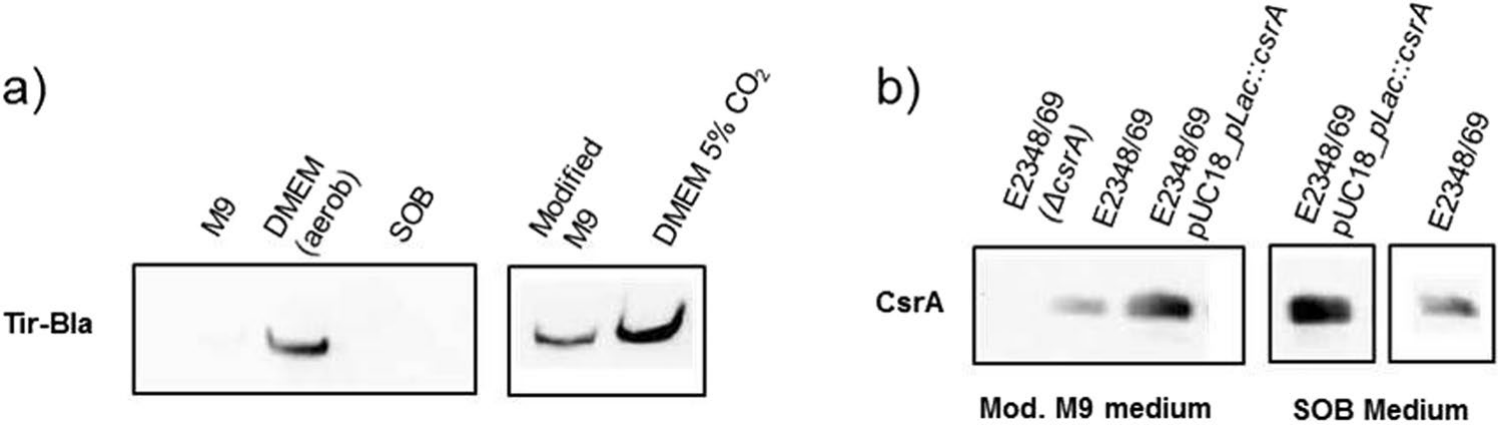
(**a**) CsrA and Tir-Bla expression in different growth media: Western blot analysis of the Tir-ß-lactamase (Tir-Bla) fusion protein from whole cell lysates under different growth conditions at an OD_600_ of ~1. The growth of EPEC in DMEM under microaerobic conditions was used as a positive control for Tir-Bla synthesis (right). (**b**) Levels of CsrA in different strains and growth media at an OD_600_ of ~1 determined by Western blots. Blots were cropped. Full length blots are shown in Fig. S4.

In order to assure that the EPEC metabolome and transcriptome were captured under conditions that also promote expression of virulence-relevant pathogenicity factors, the growth media were optimized for the expression of the most important EPEC virulence factors, e.g. the pathogenicity island LEE1-encoded T3SS. Expression of the Tir-β-lactamase (Tir-Bla) fusion protein was used as a proxy for expression of the virulence-associated T3SS genes. The Tir-Bla fusion is located in the native *tir* locus on the EPEC chromosome. As the *tir* locus is controlled via the master-regulator of the LEE-PAI *ler*, expression of the Tir-bla fusion protein is dependent on LEE-inducing conditions^33^. DMEM was used as a positive control, as it served as the standard growth medium for EPEC for studying virulence gene expression^34,35^. However, its complex composition is not suitable for meta-bolome studies. To overcome this problem, alternative minimal media were tested for the expression of *tir-bla*. Figure 1a illustrates that the Tir-Bla fusion protein is produced in a modified M9 medium supplemented with 0.2% glucose and 0.05% casaminoacids, whereas no expression was detectable in standard M9 and SOB media^36,37^. A Western blot analysis further confirmed that CsrA is expressed in modified M9 medium (Fig. 1b). In addition, it could be shown that CsrA was absent in the *∆csrA* mutant, but it could be complemented by a *csrA*-encoding plasmid in which *csrA* was induced by the IPTG-inducible *lac* promoter (Fig. 1b).

To assess the influence of the CsrA protein on gene expression and the composition of intracellular metabolites of EPEC during conditions that promote synthesis of crucial virulence factors, we compared the metabolome and transcriptome of the EPEC E2348/69 wildtype and the isogenic *ΔcsrA* mutant grown in the modified M9 medium supplemented with 0.2% glucose and 0.05% casaminoacids to early stationary phase (OD_600_ of 1), as described in *Experimental Procedures* (Fig. S1). The mRNA of the bacteria was isolated from the same culture batch used for metabolite extraction, in order to enable a direct comparison of transcriptome and metabolome data.

Although a large number of more than 3700 metabolites is listed in the *E. coli*-specific ECMDB database^38^, only a small fraction can generally be detected by a given set of extraction, chromatography and mass spectrometry conditions, because of their different physical properties. In addition, only a sub-fraction of metabolites is expressed under given biological conditions. A combination of three different approaches, using liquid chromatography-mass spectrometry (LC-MS) in negative (62) and positive mode (51) and gas chromatography-mass spectrometry (GC-MS) (46), and strict rules for metabolite assignment led to the identification of 159 metabolites (Dataset S1). These 159 metabolites were identified via matching through our measured in-house library or identified by matching the MS2 spectrum to available online databases. Unknown features (positive mode 1056, negative mode 515, total 1571, Supplementary Fig. S3A,B) from LC-MS measurements (Dataset S1) were defined by filtering to the percentage of standard error (≤15%), the error of fold change (≤15%) and the corrected p-value (≤0.05). From the identified metabolites, 30 were significantly up-regulated and 67 significantly down-regulated |fold change (fc)| ≥ 1.5; corrected p-value ≤ 0.05) between the knockout and the wildtype strain (Dataset S1 159 metabolites, metabolites identified by pure chemical standards are marked red). In parallel, RNA sequencing revealed that of 4159 profiled transcripts, 97 were significantly up-regulated and 36 significantly down-regulated in the *∆csrA* mutant compared to the EPEC wildtype, when a |log2fc| ≥ 2.0 and a corrected p-value of ≤0.05 in gene expression were taken as cut-off (Dataset S2 and Supplementary Fig. S4).

**Figure 2:**
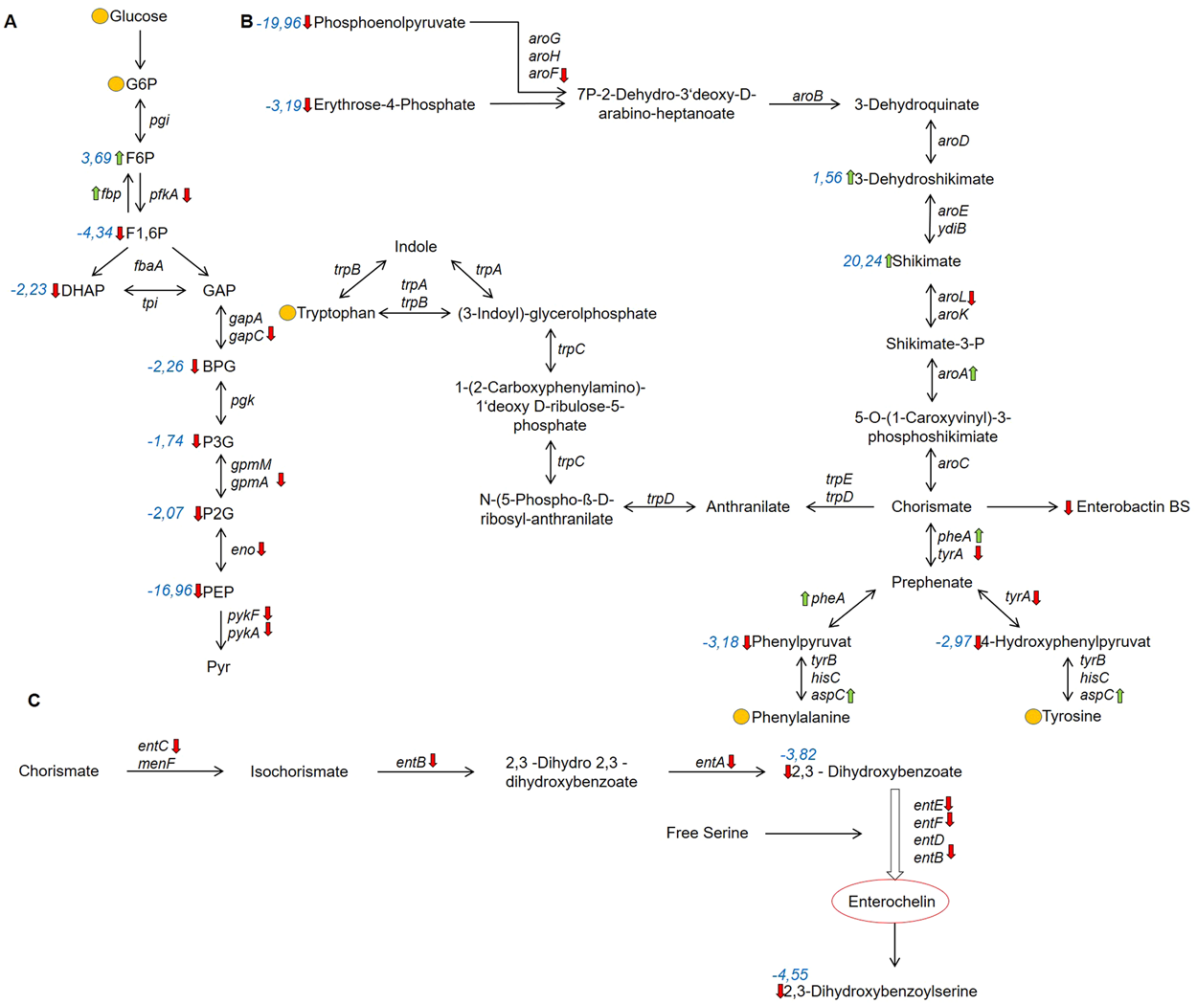
Influence of CsrA on metabolic pathways of EPEC. Regulation of glycolysis (**A**), aromatic amino acid synthesis (**B**) and enterochelin synthesis (**C**) in the EPEC Δ*csrA* mutant. Red arrows indicate downregulation, green arrows indicate upregulation, and yellow circles indicate unchanged levels in the ΔcsrA knockout strain compared to the wildtype. Quantitative fold changes and corrected p-values are listed in the Supplementary Dataset S1. Metabolites: glucose-6-phosphate G6P, fructose-6-phosphate F6P, fructose 1,6-bisphosphate F1,6P, dihydroxyacetonphosphate DHAP, glyceraldehyde-3-phosphate GAP, bisphosphoglycerate BGP, 3-phosphoglycerate 3PGA, 2-phosphglycerate 2PGA, phosphoenolpyruvate PEP, pyruvate pyr. Fold changes of significant regulated metabolites are shown in blue. Genes for enzymes: Glucose-6-phosphate isomerase pgi, fructose-1,6-bisphosphatase fbp, Phosphofructokinase pfkA, fructose bisphosphate aldolase fbaA, triose phosphate isomerase tpi, glyceraldehyde 3-phosphate dehydrogenase-A gapA, glyceraldehyde 3-phosphate dehydrogenase C gapC, Phosphoglycerate kinase pgk, Phosphoglycerate mutase M gpmM, Phosphoglycerate mutase A gpmA, enolase eno, pyruvate kinase I pykF, pyruvate kinase II pykA, 2,3-Dihydro-2,3-dihydroxybenzoate dehydrogenase entA, Asochorismatase entB, Isochorismate synthase entC, Enterochelin synthase component D entD, Component of 2,3-dihydroxybenzoate-AMP ligase entE, Enterochelin synthase component F entF, Component of isochorismate synthase menF, 3-deoxy-D-arabino-heptulosonate-7-phosphate synthase aroG, Component of 2-dehydro-3-deoxyphosphoheptonate aldolase aroH/aroF, 3-Dehydroquinate synthase aroB, 3-Dehydroquinate dehydratase aroD, Shikimate 5-dehydrogenase aroE, shikimate dehydrogenase ydiB, shikimate kinase II aroL, shikimate kinase I aroK, 5-enolpyruvyl shikimate-3-phosphate synthase aroA, chorismate synthase aroC, bifunctional chorismate/prephenate dehydratase pheA/tyrA, anthranilate synthase component trpE/D, bifunctional: N-(5-phosphoribosyl)anthranilate isomerase and indole-3-glycerolphosphate synthase trpC, Component of histidinol-phosphate aminotransferase hisC, Aspartate aminotransferase aspC.

The most striking differences between the EPEC wildtype and *∆csrA* mutant strain relate to the central carbon metabolism, the aromatic amino acid and nucleoside/nucleotide metabolism and crucial membrane-associated virulence factors. Using polar extraction methods for metabolites and a combined analysis of GC and LC-MS samples, it was possible to detect all metabolites from the Embden Meyer Parnas (EMP) pathway except pyruvate (Pyr) and glyceraldehyde-phosphate (GAP). Glycolysis is strictly regulated at one specific point in the EMP pathway **(**Fig. 2A, Dataset S1): A strong accumulation of fructose-6-phosphate (F6P) could be detected with a fold change of 3.69 at a corrected p-value (pval) of 3.56*10^−2^, whereas the downstream metabolite fructose 1,6 bisphosphate (fc –4.34, pval 6.48E*10^−3^) and all following metabolites of the EMP pathway were decreased in the *∆csrA* deficient strain (Fig. 2A, Dataset S1). On the transcriptome level, the enzymatic reaction of F6P to F1,6P was controlled correspondingly: the transcript level of fructose 1,6-bisphosphatase (Fbp) was increased in the *∆csrA* mutant compared to the wildtype, whereas phosphofructokinase (PfkA) mRNA was reduced (Dataset S2). This demonstrated that absence of CsrA leads to a drastic change of the phosphofructokinase at the swivel point of regulation in the glycolysis (Fig. 2A). In parallel, a strong accumulation of oligosaccharides from dihexoses up to heptahexoses (polysugars) was observed in the *ΔcsrA* strain (Table 2, Dataset S1). These sugars are intermediates of the glycogen synthesis. In line with this observation, the entire glycogen synthesis operon (*pgm, glgP, glgC, glgA*) was upregulated in the absence of CsrA **(**Dataset S2). Notably, the level of ADP-glucose, the key intermediate of glycogen synthesis, was decreased (Dataset S1), probably due to its permanent use in the upregulated pathway. In contrast, all following metabolites of lower glycolysis starting from F1,6 P as well as the corresponding transcripts *gapC, pgk, gpmA, pykA, pykF* were downregulated in the *ΔcsrA* mutant (Fig. 2A, Dataset S1, S2). Corresponding metabolite changes could also be observed for the downstream citric acid cycle, where seven out of eleven metabolites could be detected (Fig. S7). All identified metabolites ((iso)citric acid, 2-oxoglutarate, succinate, fumarate, malate) were significantly downregulated in the *∆csrA* knockout except acetyl CoA. Most mRNAs of the involved enzymes from the citric acid cycle (TCA) except of succinate dehydrogenase operon (*sdhABCD*) were not downregulated, indicating that the reduction of TCA metabolites results mainly from downregulation of the glycolysis enzymes.

**Table 2:** Identified oligosaccharide metabolites and their change in abundance in the Δ*csrA* mutant strain compared to the wild type.

Metabolite	Method	Fold change	Corrected p-value
Disaccharide	GC	4.81	4.79*10^−6^
Trihexose	GC	5.36	1.05*10^−2^
Trihexose	LC-positive	2.45	3.13*10^−8^
Tetrahexaose	LC-positive	2.25	4.59*10^−11^
Pentahexaose	LC-positive	2.35	5.62*10^−14^
Hexahexaose	LC-positive	3.28	4.49*10^−8^
Heptahexose	LC-positive	3.34	6.26*10^−12^
Octahexose	LC-negative	DNQ	DNQ
Nonahexose	LC-negative	DNQ	DNQ

DNQ: detected but not quantified.

In addition to the central carbon metabolism, loss of CsrA had a pronounced effect on anabolic pathways in EPEC. As illustrated in Fig. 2B, several metabolites of the aromatic amino acid biosynthesis pathway differed significantly between the *∆csrA* and the wildtype strain. Derived from sugar metabolism, the pools of phosphoenolpyruvate and erythrose-4-phosphate were downregulated in the knockout mutant. In addition, a 20-fold increase was found for shikimate (pval 5.47*10^−4^), an intermediate of the central pathway for aromatic amino acids biosynthesis **(**Dataset S1, Fig. 2B). This can be explained by the strong reduction of the mRNA encoding the shikimate-kinase AroL (fc –2.69, pval 5.70*10^−18^). AroL adds a phosphate group to the 3-hydroxy group of shikimate using adenosine triphosphate (ATP) as a substrate. Interestingly, the mRNA for AroA (fc 2.54, pval 3.28*10^−15^), which catalyzes the reaction from shikimate-3-phosphate to 5-*O*-(1-carboxyvinyl)-3-phosphoshikimate, was upregulated. Thus, it is possible that the downregulation of *aroL* in the *ΔcsrA* mutant led to an accumulation of shikimate and 3-dehydroshikimate, thereby depleting the aromatic amino acid biosynthesis downstream.

While chorismate and prephenate, intermediates of the pathway to phenylalanine and tyrosine synthesis, were not detected, phenylpyruvate (fc –3.18, pval 4.08*10^−3^), the precursor to phenylalanine, and 4-hydroxyphenylpyruvate (fc – 2.97, pval 8.50 * 10^−4^), the precursor to tyrosine, were identified and found to be significantly lower in the *∆csrA* mutant in comparison to wildtype (Dataset S1, Fig. 2B). Also mRNA levels coding for tryptophanase were reduced in the *∆csrA* knockout mutant. This explains that a lower level of indole-3-carboxylate (fc –1.87, pval 2.01*10^−3^) and a small, non-significant decrease of tryptamine (fc –1.54, pval 3.83*10^−1^) were detectable in the metabolome analysis (Dataset S1, S2). Hence, CsrA seems to mostly affect the upper part of the shikimate pathway, especially the formation of shikimate-3-phosphate, which led to an accumulation of shikimate, whereas the intermediates of phenylalanine and tyrosine synthesis were downregulated. Despite this influence, no changes in the levels of phenylalanine, tyrosine and tryptophan were detected, indicating the aromatic amino acid pool was balanced by corresponding uptake systems.

Our transcriptome analysis further revealed a pronounced reduction of transcripts encoding enzymes for the biosynthesis of enterochelin, a high affinity siderophore that chelates and acquires iron for *E. coli* and other related bacteria^39^, in the *∆csrA* mutant. Synthesis of enterochelin starts with chorismate and is closely linked to the shikimate pathway. The strongest influence of CsrA was observed for *entB*, *entA* and *entE* (fc < − 3.5), whereas *entC* and *entF* were only moderately reduced (fc < −2,5) (Dataset S2, Fig. 2C). The genes *entC*, *entE*, *entB* and *entA* are contained in a single operon in EPEC. This result was further confirmed by the metabolome analysis, in which two central intermediates and/or degradation products in the biosynthesis of enterochelin, 2,3 dihydroxybenzoate and 2,3 dihydroxybenzoylserine were found to be strongly reduced (fc –3.82, pval 1.31*10^−8^ and fc −4.55, pval 1.31*10^−8^, respectively; Dataset S1, Fig. 2C). Electrophoretic mobility shift assays revealed complex formation of purified CsrA with *entC* mRNA leader, indicating that the influence of CsrA on the expression of the *ent* operon is direct (Fig. S9).

Another striking set of changes induced by the *∆csrA* knockout mutant concerned the accumulation of nucleosides (e.g. uridine, guanosine). In contrast, the metabolic pools of other nucleotides such as UMP, AMP, ADP were significantly reduced in the *ΔcrsA* strain (Dataset S1). Nucleosides and bases are provided by nucleotide salvage pathways. The abundance of the transcripts of the enzymes involved in nucleotide salvage pathways like the cytidine deaminase *cdd*, the pyrimidine specific hydrolase *rihB* and the thymidine dephosphorylase *deoA* were downregulated, whereas the acid phosphatase/phosphotransferase *aphA* mRNA level was slightly enhanced (Dataset S2). In addition, also partial changes in the metabolites linked to nicotinate and riboflavin metabolism were visible. The redox-equivalents NADP and NAD were significantly reduced, and also the intermediate of the synthesis and salvation pathway nicotinamide ribotide (NMN) was strongly depleted. In contrast, higher levels of the precursors nicotinate and nicotinamide were detected in the knockout strain. In addition, the concentrations of the important cofactors FMN and FAD were significantly reduced in the *∆csrA* mutant (Dataset S1).

**Figure 3:**
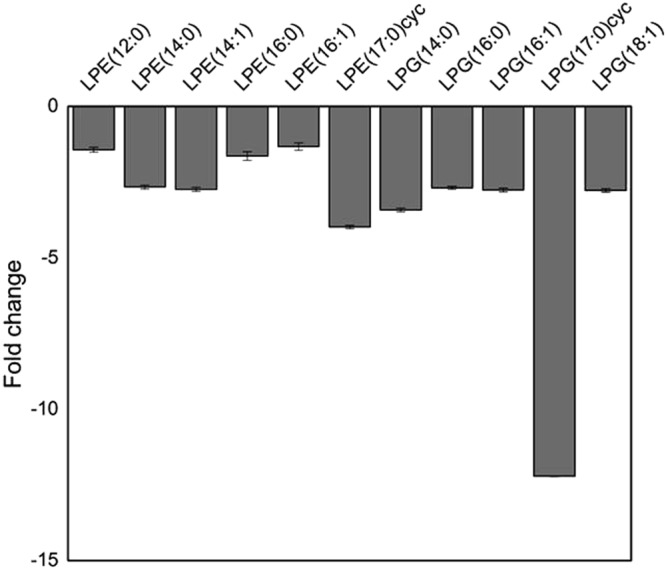
Bar diagram of lysolipid levels of EPEC. Fold changes in abundance for lysoPE and lysoPG species in the Δ*csrA* strain compared to the EPEC wild type. Data represent averages from positive and negative measurements. Only significant values were used to create this figure (corrected p-value ≤ 0.05). Error bars represent the average relative error of the fold change. All log2fold changes and corrected p-values are listed in Table S3.

A strong influence of CsrA was observed for different lipid pools (Table S3). The detection of phosphatidylethanolamine (PE) and phosphatidylglycerol (PG) lipids, important components of the inner membrane of *E. coli*^40,41^ was generally limited, as the extraction and separation methods of our study were optimized for species with medium polarity. However, various PE, lysoPE and lysoPG species (intermediates in the metabolism of lipids) were detectable and found to be significantly reduced in the *∆csrA* knockout (Fig. 3, Table S3, Dataset S1). In particular, the cyclic lyso-PE 17:0 and the cyclic lyso-PG 17:0 showed the strongest reduction of all detected lyso-lipid species. In agreement, the transcript abundance of the enzyme catalyzing the modification, the cyclopropane fatty acid synthase (*cfa*) (fc – 1.66; p-val 1.31 E-03; Dataset S2), was reduced. However, no CsrA binding was observed with the *cfa* ‘5-UTR (Fig. S7).

The RNA-seq analysis further revealed that many virulence-associated traits, in particular those associated with the bacterial cell envelope, were strongly upregulated in the absence of CsrA (Dataset S2). The mRNA levels coding for the structural components of the T3SS secretion system (*escF, escR, escT, escN, escU, sepQ, escV*), as well as the mRNAs for the secretion factors *espH, espF, map* were significantly upregulated in the *∆csrA* mutant. Also the transcript level of the adhesin intimin (Eae) and the equivalent translocated intimin receptor (Tir), which are necessary for EPEC to attach to the host cell surface, were strongly enriched. Upregulation of these LEE-encoded genes is promoted by the induction of the transcriptional activator Ler (log2fc = 1.96; Dataset S2). Moreover, the transcript level of the Ler-induced positive regulator of GrlA was significantly elevated in the absence of *csrA*.

The highest positive changes for transcripts (up to 50 fold) were reached for mRNAs encoding building blocks of colanic acid and the corresponding membrane export proteins (*wza, wzb, wzc, wzxC*) (Dataset S2). Colanic acid, also known as the M-antigen, is responsible for forming a protective capsule around the bacteria, which enhances their ability to survive in an acidic environment. To test whether upregulation of these operons led to enhanced exopolysaccaride (EPS) production and secretion, the glucose content was determined from the EPS matrix through an anthrone/H_2_SO_4_ based photometric assay. Optical density-normalized data revealed that EPS of the stationary phase EPEC *∆csrA* cells contained seven times more glucose (70.19 µg/ml ± 11.49 µg/ml) than the wildtype strain (10.66 µg/ml ± 1.84 µg/ml). In addition, the colanic acid content was estimated by an assay that quantifies the color reaction of fucose with cysteine hydrochloride. Fucose, also known as methylpentose, is a methylated desoxy-sugar and a specific component of colanic acid^42^. The fucose concentration was determined from liquid culture and normalized to the optical density. Under these conditions, the *∆csrA* strain produced three times more colanic acid (1016.1 µg/ml ± 362.4 µg/ml) than the wildtype strain (311.3 µg/ml ± 184.8 µg/ml), supporting our expression data. Finally, an electro mobility shift assay (EMSA) was applied to probe a direct binding of CsrA_His6_ to the *wza* 5’ UTR. However, no higher molecular weight complex was detectable, indicating that the influence of CsrA on expression of the *wza* operon is indirect (Fig. S5).

In parallel to upregulation of the colanic acid capsule genes, the abundance of *hdeABD* transcripts encoding acid resistance proteins was strongly reduced (*hdeA* fc –14.36, *hdeB* fc –13.18, *hdeD* fc –13.90).

**Figure 4:**
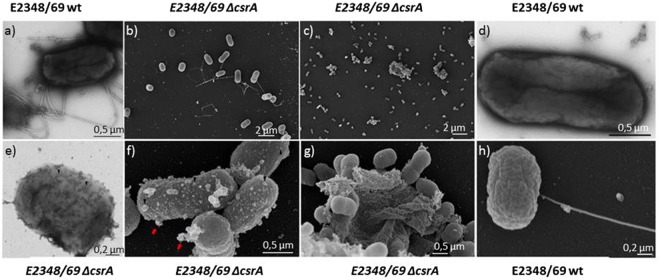
Phenotypic changes of the ΔcsrA mutant. Transmission electron microscopy (TEM) pictures of negative stained EPEC (E2348/69) wildtype (**a**,**d**) and the isogenic E2348/69 ΔcsrA mutant (**e**). Field electron microscopy (FEM) pictures of EPEC (E2348/69)(**b**,**h**) and the E2348/69 ΔcsrA mutant (**c**,**f**,**g**).

Next, we studied the phenotype and cell morphology of the EPEC wildtype and mutant by transmission electron microscopy (TEM) and field emission scanning electron microscopy (SEM). The *ΔcsrA* mutant lost the typical rod-shaped cell morphology of the wildtype (Fig. 4). The *ΔcsrA* strain had a more coccus-like structure, which is atypical for exponentially grown *E. coli*, and pili as well as flagellae were absent. Also the cell size of the *∆csrA* mutant was significantly smaller than that of the wildtype strain (1.18 ± 0.18 µm vs. 1,45 ± 0.07 µm; p-value = 1.40E-11; Dataset S1). Moreover, transcripts of the corresponding *fim* and *flhDC* operons were downregulated. The cells also tended to aggregate and formed cluster-like structures and slimy colonies. Interestingly, on the surface of the outer membrane of most *ΔcsrA* mutant cells multiple vesicle-like structures were exposed, which were absent in the EPEC wildtype strain (Fig. 4). These structures were also visible by TEM (Fig. 4a,d).

## Discussion

This study provides a comparative analysis of the metabolome and corresponding transcriptome of an entero-pathogenic EPEC strain E2348/69 and its isogenic *∆csrA* mutant derivative. The untargeted analysis of the metabolome and transcriptome reveals the complexity of the CsrA regulon, its multiple effects on various metabolic pathways and a tight link with EPEC virulence-relevant components. A limitation of the study is that the *∆csrA* mutant exhibits a slower growth rate than the wild type (in accordance with previous studies). Therefore, we cannot exclude that impaired growth may cause changes in expression that are not simply a reflection of the regulation by CsrA, but may result from altered growth.

It is known from previous studies that *csrA* mutants in *E. coli* K-12 strains and *Salmonella* are genetically instable and rapidly evolve suppressor mutants in the glycogen synthesis operon (*glg*)^32,43^. However, glycogen staining of the *∆csrA* mutant did not reveal major phenotypic variations of the colonies (Fig. S2), implying that the *csrA*-deficient EPEC E2348/69 strain is rather stable.

In our study, expression of the glycolysis pathway genes was found to be significantly affected in the *∆csrA* mutant. A decrease in most glycolytic activities, especially the phosphofructokinase, accumulation of metabolites in the upper glycolysis and glycogen pathways, and depleted TCA metabolites could also be detected in the metabolite pools of non-pathogenic *E. coli* K-12, harboring a partial deletion of the last 10 amino acids of the CsrA protein, and in the CsrA-attenuated commensal strain Nissle 1917^8,9,12^. Also recent results of a CLIP-seq analysis indicated that glycolysis is directly regulated by CsrA due to stabilizing of the *pfkA* mRNA. Additional changes in glycolysis, citric acid cycle and gluconeogenesis may be indirectly or directly influenced by CsrA^8^. We observed an upregulation of *sdhABCD* transcripts (log_2_ fc 1.32–1.70, Dataset S2), which is in line with data from Sowa *et al*., who reported weaker effects in the same direction (log_2_ fc 0.25–1.17). Notably, Sowa also found that the *sdhD* coding sequence in *E. coli* contains a regulatory element which directly interacts with CsrA and effects also translation of the following gene *sdhA*^18^.

Interestingly, a sequestration of CsrA via overexpression of the CsrA regulatory sRNA CsrB led to increased levels of phenylalanine and PEP^44–46^. In contrast, the complete *∆csrA* knockout strain used in our study led to reduced PEP, downregulated aromatic amino acid synthesis pathways but not to significant changes of the metabo-lite concentration of the aromatic amino acids including phenylalanine. Nevertheless McKee *et al*.^44^ used a CsrB overexpression to surpress CsrA function. Overexpression of CsrB does not completely inhibit the regulatory function of CsrA like a complete deletion. Also the growth media and strains differ from our experimental setup, which could explain the different observations compared to our results. A flux analysis showed that the reduced flux through the EMP was not diverted to glycogen synthesis, but redirected towards the PP pathway^9^. Our findings that the Csr system controls several pathways of the central carbon metabolism like glycolysis, gluconeogenesis, glycogenesis and the TCA cycle are in line with those reported for non-pathogenic laboratory, commensal and industrial strains of *E. coli*^9,12,44,47^ and for other enteric organisms such as *Y. pseudotuberculosis*^48^. This indicates that the influence of CsrA on multiple steps of the central carbon metabolism is conserved between pathogenic and non-pathogenic *Enterobacteriaceae*. An impaired glycolytic flux may contribute to the strong growth phenotype of *∆csrA* mutants of different members of this family. This reinforces previous data indicating that the Csr system improves the competitiveness of *E. coli* in the use of carbon sources. Additional data of this study extend this role by showing that CsrA also influences the metabolism of many other biomolecules, and this influence is likely to improve growth efficiency. The strong imbalance in the metabolic pools of the nucleosides/nucleotides, redox equivalents and cofactors are likely to contribute to the growth defect of the *ΔcsrA* mutant, considering their importance for DNA replication, mRNA synthesis and energy production. Also changes in the lipid composition were visible, in particular among lyso-PE and lyso-PG species. It is possible that changes of the lipid pool induce morphological changes, which may influence lipid bilayer stiffness, membrane fluidity and stability, ion channel and membrane protein/complex activities^49^. However, given the limited knowledge on these lipid classes, a more detailed functional analysis of the EPEC’s lipidome is required to gain more information about the physiological consequences. Potts *et al*. showed that CsrA regulates genes related to diverse functions of the cell envelope by direct binding to the 5′-UTR of the extra-cytoplasmatic stress response sigma factor σ^E^ (RpoE) and its membrane-bound antagonist RseA^8^. As a consequence, *∆csrA* mutants are less resistant to envelope stresses. Nevertheless,*rpoE* expression was not significantly altered in the study of Potts *et al*., consistent with our observation (Dataset S2**)**. Also changes in the LPS structure and disruption of outer membrane asymmetry can increase sensitivity to stress. Potts *et al*. did not find any link of CsrA mediated changes in lipopolysaccharid synthesis and remodeling, but a marked decreased expression of *ompF* and *phoE*^8^. In contrast to the results of Potts *et al*., we could not observe significant changes in the expression levels. As the regulation of the σ^E^ factor cross-interacts with several regulatory systems in dependence of the environmental conditions, the different experimental setups may be responsible for the distinct regulatory patterns. In total, the results of the previously published studies by Potts *et al*. and Sowa *et al*. show similar effects of CsrA to the transcriptional landscape of *E.coli*. Nevertheless differences in some expression levels could be explained due to the different methods (CLIP-Seq., Ribosome Profiling) and bioinformatics analysis pipeline. In addition, we used virulence-optimized minimal media for our experimental setup, whereas Potts *et al*. used LB full media for bacterial growth. Also strain specific differences could influence the observed results of the different studies.

This study also documents that the Csr systems regulates the biosynthesis of aromatic amino acids, siderophores, and colanic acids in EPEC. As iron is usually limited and an essential trace element during infection, iron uptake is generally strongly linked with and essential for EPEC virulence^50^. The influence of CsrA on the entire *ent* operon suggests that this regulator affects the expression of a transcription factor or the stability of the *entABCDEF* mRNA. The formation of a CsrA-*entC* mRNA complex by EMSAs (Fig. S9) indicated that CsrA influence on the *ent* operon expression is direct. The effect of CsrA on enterochelin expression and on other siderophore biosynthesis pathways of *E. coli* has not been investigated in detail before. Nevertheless Potts *et al*. also observed an influence of CsrA on the iron metabolism in an enrichment analysis for the genes involved in enterobactin biosynthesis and iron transporters, which was not visible in the CLIP-seq analysis, and therefore probably an indirect effect of CsrA regulation^8^. Anyhow the major regulator of iron homeostasis *fur* was not regulated in the study of Potts *et al*., consistent with our transcriptome analysis. In contrast, serval mRNA involved in iron storage (*ftnB*, *dps* and *bfr*)^8^ were strongly repressed in the study of Potts *et al*., but not affected in our experimental setup. It is well known that iron metabolism is closely linked to the carbon metabolism of pathogens. For instance, expression of many virulence and metabolic genes are under control of the cAMP receptor protein CRP, a global regulator of genes for carbon source utilization in the absence of glucose and the ferric uptake regulator Fur^8,51^. Moreover, several iron uptake systems and proteins controlling iron homeostasis have been identified in transcriptome analysis as part of the CsrA regulon in other pathogens^26,52,53^ Our study now provides metabolomic evidence that the Csr system exerts a link between carbon metabolism and iron uptake to optimize fitness and virulence during infection by its influence on the aromatic amino acid biosynthesis pathway, which includes the biosynthesis of one of the most potent bacterial iron chelators.

One of the most striking effects associated with the loss of CsrA was the up-regulation of the colanic acid synthesis genes. These genes are common in *Enterobacteriaceae*^54^ and normally only expressed under acidic stress conditions^55,56^. Colanic acid is one component of a distinct type of EPS, which forms a negatively charged polysaccharide capsule (M-antigen) on the surface of pathogenic *E. coli, Salmonella* and other *Enterobacteriaceae*^57–59^. The highly complex EPS matrix structure and its cell association are not well defined, as the included oligosaccharides cannot be easily distinguished by available analytical methods, but it is known that they play an important role for bacteria living in biofilms, which were shown to be repressed by CsrA^57,60^. Recent work has also demonstrated that colanic acids bound to the outer membrane maintain the membrane bioenergetics and integrity and the protein motive force, in particular during envelope stress and under metal limiting conditions^61,62^. This occurs in cooperation with the phage shock response (Psp) encoded by the *pspABC* operon, which is also CsrA-dependent (Dataset S2). Moreover, they affect the susceptibility of membrane-damaging agents as well as biofilm formation, known to influence pathogenicity^61,63,64^. As the EPS layer has a protective function against acidic stress^65^, expression of acid resistance proteins may not be necessary in the *∆csrA* knockout strain with the enlarged EPS layer. In line with this, we observed a strong downregulation of transcripts encoding enzymes for cyclopropane fatty acids synthesis and acid resistance proteins that are known to protect the bacterial cell against acid shocks^41,66^. Nevertheless, a CsrA_His6_ EMSA with the 5’UTR of the *wza* gene did not provide evidence of a direct interaction between CsrA and the colanic acid synthesis operon (Fig. S5), which implies that the strong upregulation of the colanic acid genes observed in the transcriptome data set is due to an indirect effect of CsrA on colanic acid synthesis and export or to the regulation of the upstream regulator of the colanic acid synthesis *rcsA*. Further experiments are required to elucidate whether regulation of colanic acids by CsrA is caused through another regulatory component or influenced by adjacent metabolic pathways. In addition, no binding of CsrA to the *cfa*-5’UTR fragment was detectable, indicating that CsrA influence on *cfa* expression is indirect (Fig. S8). Secondary effects of the *csrA* deletion are most probably responsible for the strong effects on the cyclopropane lysolipid level in the metabolome and the observed changes of the *cfa* transcript level in the transcriptome.

Our data further revealed that the Csr system governs also the expression of the most important virulence factors of EPEC. Among the most strongly upregulated virulence genes in a *∆csrA* mutant are those of components of the crucial T3SS and effectors encoded on the pathogenicity island LEE1^24^. They are required for the attachment and reorganization of the cytoskeleton of attached host cells. LEE consists of five polycistronic operons (LEE1-5) coding for structural components of the T3SS, several secretion factors and transcriptional regulators of the system. Control of LEE expression is highly complex and is affected by a plethora of environmental factors (e.g. temperature) and multiple transcriptional activators, of which Ler is known to strongly activate all LEE1-5 units and the monocistronic genes *escG, map* and *grlA* in the *∆csrA* mutant^67,68^. Also GrlA, which influences *ler* expression in a positive feedback loop^69^ was elevated^68^. In contrast to our study, Bhatt *et al*. demonstrated that a loss of the *csrA* gene in the identical EPEC strain led to a reduction of LEE4-encoded transcripts and abolished formation of actin pedestals on epithelial cells^23^. Consistent with this result, secretion of the translocators EspA, EspB and EspD and the level of the *espADB* mRNA determined by real-time qRT-PCR were substantially reduced. Strikingly, this influence was not mediated by the regulators GrlA and Ler, it occured through direct interaction of CsrA to the *espADB* leader mRNA^23^. However, in our experimental setup *espADB* transcript levels were not differentially expressed in the *∆csrA* mutant strain. One obvious difference between the experimental settings was the use of different growth media, and the *csrA* mutant alleles were distinct in both studies. Interestingly, the influence of a *csrA* deletion is different in the close relative strain EHEC O157:H7. Disruption of *csrA* led to an almost uniformly up-regulation of the entire LEE, including the master regulator gene *ler* in EHEC^70^. This suggests that the influence of CsrA on its target genes is strongly dependent on the status of the metabolism and the availability of nutrients/ions (Fig. 1), as reported in the literature^7^, and that it differs between EPEC and EHEC strains. A comparative analysis shows that CsrA also impacts T3SS expression in other enteric pathogens, e.g. *Y. pseudotuberculosis*, *Shigella flexneri* and *Salmonella enterica* serovar *typhimurium*^19,71–73^. A fine-tuned control of these highly energy consuming molecular machines in tight coordination to accessible energy sources appear a prerequisite to maintain the bacterial fitness in a hostile environment during the infection.

In summary, this global metabolome and transcriptome analysis addressing the role of CsrA in EPEC under virulence factor-inducing conditions revealed multiple affected metabolites and pathways, which have not yet been linked to the carbon storage system. The results provide a starting point for a functional validation by proving direct mRNA-CsrA interactions and a more in-depth analysis of the influence of CsrA on the bacterial lipidome.

## Experimental procedures

### Strains and plasmids

The EPEC wildtype strain E2348/69 was used in this study. The *csrA* deletion mutant was constructed by a standard procedure using the lambda red recombinase system as described before^23^. Endogenous *csrA* was replaced by a kanamycin cassette amplified from vector pKD4^74^. Nucleotide sequences were amplified from the E2348/69 genome. Primer sequences used in this study are shown in Table S1, and strains and plasmids are listed in Table S2 in the supplemental materials.

### Media and growth conditions

Bacteria were grown in either Super Optimal Broth (SOB) full media^75^, modified M9 media (MgSO_4_ 1 mM, CaCl_2_ 0.6 mM, FeSO_4_*7H_2_O 12.5 µM, NH_4_Cl 1 mM, Fe(NO_3_)_3_*9H_2_O, NaCl 100 mM, KCl 5 mM, NaH_2_PO_4_*H_2_O 1 mM, Na_2_HPO_4_ 33 mM, KH_2_PO_4_ 22 mM) or MOPS minimal media^76^ supplemented with 0.2% glucose (w/v) and either 0.05% or 0.02% (w/v) casaminoacids at 37 °C, 160 rpm in a standard incubator. The composition of the modified M9 medium was optimized for virulence gene expression. For metabolome and transcriptome sample preparation, an overnight pre-culture was used to inoculate the main culture at a start OD_600_ of 0.05. At least six independent biological and two technical replicates per biological replicate were made.

### Growth curve and OD_600_-biomass correlation

Overnight cultures of EPEC strains grown in SOB medium were used to inoculate 150 ml M9 medium at a start OD_600_ of 0.05. Three biological replicates per strain were used to examine bacterial growth. Samples for OD_600_ measurements were taken every hour until the OD_600_ reached 0.5, and then every 30 min to follow exponential growth. For each time point, three technical replicates for each biological replicate were measured. At least three measurements were performed after cultures reached the stationary phase. Biomass-OD_600_ correlation was determined by taking 10 ml samples of each strain during exponential growth until the culture reached the stationary phase in at least three independent biological replicates. At three different time points, 9 technical replicates were sampled to calculate the OD_600_-dry mass correlation. Bacterial cells were pelleted at 10,000 x g for 5 min, and the medium was discarded. Cell pellets were dried over night at 80 °C in a drying oven to eliminate the remaining liquid and the bacterial dry mass was measured.

### Metabolome sample preparation

Bacterial cells were grown until an OD_600_ of 1 was reached. Then, 25 ml of cell suspension were directly plunged in 25 ml of 60% ice cold methanol to quench metabolism. Cells were harvested at 4 °C for 5 min at 11,000 x g. The supernatant was discarded, and the cell pellets were directly shock frozen in liquid nitrogen. Metabolite extraction for liquid chromatography coupled to mass spectrometry (LC-MS) was performed by resuspending the cell pellet with 600 µl of pure, ice-cold methanol. The cell suspension was shock-frozen again in liquid nitrogen, and 600 µl of deionized water was added. Further metabolite extraction with repeating freeze-thaw-sonification cycles followed, as described previously^77^. Initial steps of metabolite extraction for gas chromatography coupled to mass spectrometry (GC-MS) and for LC-MS were identical. One additional step was added at the end for GC-MS samples that consisted of the addition of 1 ml chloroform and the separation of the unpolar phase from the polar phase.

### LC-MS conditions and data analysis

For LC-MS analysis, 3 µl per sample were injected. For each of the strains (conditions), 6 biological replicates (6 separate cultures) were used, and of each culture two independent extractions were performed (2 technical replicates for each biological replicate) resulting in a total amount of 12 samples per conditions. Pool samples of each condition and a whole pool sample of all conditions were used for feature identification. All samples, together with the pool samples and a quality control sample (containing 2-methoxybenzoic acid, biochanin A, *trans*-ferulic acid, 3-indoleacetonitrile, indole-3-carboxaldehyde, kaempferol, kinetin, *p*-coumaric acid, L-(+)-ß-phenylglycine, phlorizin hydrate, rutin trihydrate, indole-3-acetyl-L-valine) were randomized before batch measurement. Quality control samples were compared at the beginning and at the end of a batch run to check for shifts in the retention time (RT) and the exact masses of the identified metabolites. For further data normalization, the LC extraction buffer was spiked with three internal standards (nortriptyline 200 µg/ml, trimethoprim 200 µg/ml, glipizide 600 µg/ml). The resuspension buffer (50% acetonitrile/water with 0.1% formic acid) was further spiked with caffeine 1 mg/ml and naproxen 8 mg/ml. Ultra-high performance liquid chromatography was performed on a Dionex Ultimate 3000 UPLC by using a reverse phase column (Phenomenex Kinetex 1.7 µ C18 150 × 2.1 mm diameter column) with a flow rate of 300 µl/min. Water/acetonitrile were used as mobile phases, with A: water + 0.1% formic acid and B: acetonitrile + 0.1% formic acid. A linear gradient was used: Start: 1% B, 2 min: 1% B, 20 min: 100% B, 25 min: 100% B, 30 min: 1% B. Whole metabolome samples were analyzed by quadrupole time-of-flight mass spectrometry on a Bruker Daltonics maXis HD QTof instrument using electrospray ionization in positive and negative modes. Raw data of the metabolome study were exported as mzXML.data and analysed using XCMSonline [https://xcmsonline.scripps.edu/] and Bruker Daltonics DataAnalysis software. Standard parameters for XCMSonline were used for further data analysis if not specified otherwise. Feature detection was performed using the CentWave algorithm and a maximum tolerated m/z deviation in consecutive scans at 5 ppm. Retention time correction was performed using the obiwarb algorithm (1 m/z step wise). Accurate masses were obtained by internal calibration using a sodium formiate cluster and lock mass calibration.

Statistically significant regulated features were searched against the METLIN (https://metlin.scripps.edu/) metabolite database and the *E. coli* metabolome database (ECMDB, http://ecmdb.ca/). Metabolite identifications were achieved by matching the retention time, MS and MS/MS fragmentation pattern of each feature of interest to an in-house library of 600 metabolites as pure chemical standards. For lipids, one chemical standard per lipid class was used as the basis for assigning the MS2 spectra and/or comparison to online databases. Metabolites which were identified by pure chemical standards are marked as red in the Supplementary Dataset S1. Features which could not be identified with the in-house library were putatively identified by matching the exact mass and MS/MS fragmentation pattern to open-source MS/MS libraries like ECMDB and METLIN DB, or to theoretical MS/MS fragmentation patterns generated by MetFrag [http://msbi.ipb-halle.de/MetFrag/].

### Derivatisation of GC-MS samples

The polar phases of GC-MS samples were derivatized with 40 µl methoxyamine/pyridine solution (20 mg/ml w/v) for 90 min at 30 °C, while shaking at 1000 rpm. After that, 60 µl of MSTFA was added, and a further incubation step at 37 °C for 30 min at 1,000 rpm was performed. Samples were measured directly after derivatization. For further data normalization, GC-MS samples were spiked with ribitol at a final concentration of 10 µg/ml.

### GC-MS conditions and data analysis

For GC-MS analysis, 1 µl of each sample was injected. Pool samples of each condition and a whole pool sample of all conditions were generated, and were randomized in the sequence, together with the samples and blanks as described for the LC-MS measurements (see also LC-MS conditions and data analysis). At the beginning and before the end of the batch run, and also after every five samples an alkane mix (decane, undecane, dodecane, tridecane, pentadecane, octadecane, nonadecane, docosane, pentacosane, ocatacosane, dotriacontane, hexatriacontae), and the whole pool sample were injected and analyzed as a quality control. The column was washed after 10 samples by injecting cyclohexane. Samples were analyzed on a Thermo Trace Ultra GC - ITQ900MS GC-MS using a Phenomenex ZB-5MS column (30 m × 0.25 mm i.d., 0.25 µm particle size, plus 5 m integrated guard column) for metabolite separation. The used injection mode was a 1 µl split-less injection with injector ramp conditions of 70 °C to 280 °C at 14 °C/s. Gas chromatography was performed using an initial oven temperature at 70 °C for 1 min that was ramped to 330 °C at 8 °C/min. The helium flow was set to 1.2 ml/min. MS parameters were set for positive electron ionization (EI + ) at 70 eV, and full scan MS mode with an m/z acquisition range of 50-600 m/z. Raw data obtained from GC-MS experiments were exported to netCDF using the Excalibur (Thermo Fisher Scientific) software. Further data analysis and metabolite identification was performed using the latest version of MetaboliteDetector^78^.

### Statistical analysis

Data statistics were calculated using R (packages: metabolomics, pcamethods). T-test correction was performed using the False Discovery Rate correction by Benjamini-Hochberg^79^. Significant features were filtered by corrected p-value ≤ 0.05 and a maximum percentage of 20% standard error in between the replicates of the tested conditions. Identified metabolites were determined as significantly regulated with |fc| ≥ 1.5 and a significant corrected p-value ≤ 0.05. Instead of log2 fold change, we used the normal fold change to describe significant changes between deletion mutant and wildtype. For values <1, we calculated the negative reciprocal to get an equal scaling.

### Western blotting

SDS polyacrylamide gel electrophoresis (SDS-PAGE) of CsrA or the Tir-Bla fusion protein in the *∆csrA* deletion mutant and E2348/69 wildtype strain were performed following the protocol for glycine SDS-PAGE^80^. SDS-PAGE gels were transferred to a nitrocellulose membrane^81^ and incubated with the primary, polyclonal antibody against CsrA (αCsrA 1:2000) for at least 1 hour. Membranes were incubated with the proper secondary α-rabbit antibody, which was conjugated to horse radish peroxidase (HRP) (Cell signaling, 1:2000). Reactions were developed by chemiluminescence (Clarity ECL, Bio-Rad). For the detection of expressed Tir-Bla fusion protein from the EPEC reporter strain^33^, primary β-lactamase antibody (Cell signaling 1:2000) was used. Membranes were incubated with the proper secondary α-mouse-HRP antibody (Cell signaling, 1:2000).

### RNA isolation

Total nucleic acid isolation was performed by hot phenol extraction. Cell pellets were resuspended in 250 µl resuspension buffer (0.3 M sucrose, 0.01 M NaOAc, pH 4.5). Then, 250 µl of lysis buffer (2% SDS (w/v), 0.01 M NaOAc pH 4.5) were added, and the suspension was incubated for 90 s at 65 °C. 500 µl of pre-warmed phenol-water (Roth) was added and mixed. After 3 min of incubation at 65 °C, the tubes were frozen in liquid nitrogen and then centrifuged for 10 min at 11,000 × g. 300 µl of chloroform: isoamylalcohol (24:1, Roth) was added to the aqueous phase, and the solution was again centrifuged for 3 min at 11,000 × g. 1/10 Vol. of 3 M NaOAc pH 4.5 and 2.5 Vol. of 95% ethanol was added to the aqueous phase and incubated for at least 1 h at −20 °C. The nucleic acid precipitate was recovered by centrifugation (11,000 × g, 4 °C) for 30 min. The nucleic acid pellet was washed once with 70% ethanol and dried in a speedvac. Chromosomal DNA was digested using the Turbo DNaseI (Ambion) kit following the instructions of the manufacturer.

### Microscopy

Overnight cultures of the E2348/69 wildtype strain and the Δ*csrA* strain were inoculated at a start OD_600_ of 0.05 and grown to a final OD_600_ of 1.

### Negative staining of bacteria

Thin carbon support films were prepared by sublimation of carbon onto a freshly cleaved mica surface. Bacteria were negatively stained with 1% (w/v) aqueous uranyl acetate, pH 5.0, according to the method of Valentine *et al*.^82^. Samples were collected with 300 mesh copper grids, washed in TE buffer (20 mM TRIS, 2 mM EDTA, pH 6.9), distilled water and air dried. Samples were examined in a TEM 910 transmission electron microscope (Carl Zeiss, Oberkochen) at an acceleration voltage of 80 kV. Images were taken at calibrated magnifications using a line replica. Images were recorded digitally with a Slow-Scan CCD-Camera (ProScan, 1024 × 1024, Scheuring, Germany) with ITEM-Software (Olympus Soft Imaging Solutions, Münster, Germany).

### Field emission scanning electron microscopy

Samples were fixed with 2% glutaraldehyde and 5% formaldehyde in HEPES buffer (HEPES 0.1 M, 0.09 M sucrose, 10 mM CaCl_2_, 10 mM MgCl_2_, pH 6.9) kept at 7 °C for overnight. Cover slips with a diameter of 12 mm were coated with a poly-L-lysine solution (Sigma, Munich, Germany) for 5 min, washed in distilled water and air-dried. 50 µl of the fixed samples were placed on a cover slip and allowed to settle for 10 min. Cover slips were then fixed in 1% glutaraldehyde in TE-buffer (20 mM TRIS, 2 mM EDTA, pH 7,0) for 10 min at room temperature and subsequently washed with TE-buffer before dehydrating in a graded series of acetone (10, 30, 50, 70, 90, 100%) on ice for 10 min for each step. Samples were then subjected to critical-point drying with liquid CO_2_ (CPD 300 Leica, Wetzlar, Germany). Dried samples were covered with a gold-palladium film by sputter coating (SCD 500 Bal-Tec, Balzers, Liechtenstein) before examination in a Zeiss field emission scanning electron microscope Merlin (Oberkochen, Germany) using the Everhart Thornley SE detector and the inlens SE detector in a 50:50 ratio with an acceleration voltage of 5 kV. Contrast and brightness were adjusted with Adobe Photoshop CS5.

### Preparation of colanic acid

Colanic acid extraction was based on a protocol described previously^83^. 50 ml of an overnight culture of the corresponding strain was heated for 15 min at 100 °C to denature degrading enzymes. The suspension was cooled down and centrifuged at 11,000 × g at 4 °C for at least 30 min. 40 ml of supernatant was precipitated with three volumes of ice-cold ethanol. After incubation for 12 h at 4 °C, the mixture was centrifuged at 11,000 × g at 4 °C for 30 min, and the resulting pellet was dissolved in 5 ml of distilled water. Dialysis (membrane MWCO 3500 Da) was performed for 48 h against distilled water and the remaining liquid was dried in a centrifugal evaporator overnight at 4 °C. Remaining polypeptides were removed by precipitation with 5 ml of 10% (v/v) trichloracetic acid. The samples were dialyzed again for five days against distilled water and finally dried as described above. The resulting colanic acid preparation was re-suspended in 1 ml of distilled water and used for quantification.

### Quantification of colanic acid

Colanic acid quantification was performed as described^84^. The method is based on the detection of non-dialyzable fucose (6-deoxy-hexose) in a photometric assay. 10 to 100 µl of the colanic acid samples were diluted in 1 ml of distilled water and mixed with 4.5 ml sulfuric acid/water (6:1, v/v). The samples were heated at 100 °C for 20 min and then allowed to cool to room temperature. Normalization to an unspecific color reaction was performed as previously described^42^. For each sample the absorbance was measured directly at 396 nm and at 427 nm (control sample), and after the addition of 100 µl of cysteine hydrochloride (cysteine sample) at both wavelengths. The absorbance measured in the control sample was subtracted from the total absorbance in the corresponding sample with cysteine (ΔA_396_, ΔA_427_). Values of ΔA_396_-ΔA_427_ were used to correlate the fucose concentration of the sample to a standard curve ranging from a fucose concentration of 5 µg/ml to 100 µg/ml.

### Quantification of total exopolysaccharides

The protocol used was adapted from the protocol to extract and analyze exopolysaccharides (EPS) in *E. coli*^51^. Bacterial cultures were grown on an LB agar plate for 12 h at 37 °C. Total exopolysaccharides (EPS) were quantified by taking 60 mg of bacterial culture from the LB plate and resuspending it in 1 ml of sterile water. The cell concentration was determined by OD_600_ measurements. The culture was boiled for 10 min at 100 °C in a heat block to inactivate enzyme activity. Samples were centrifuged at 16,000 x g for 10 min, and the supernatant was used for the assay. The quantification assay was reported before^85^. In brief, 400 µl of each sample were mixed with 100 µl freshly prepared anthrone solution (2%) in ethyl acetate. The reaction was started by addition of 1 ml of sulfuric acid. Absorbance was measured at 620 nm after the solution cooled down to room temperature. Glucose equivalents from the EPS were quantified by using a glucose calibration curve (10 to 100 µg/ml). Normalization was performed using the optical density of the cell solutions.

### RNA isolation, strand-specific library preparation and Illumina sequencing

Total bacterial RNA of the three different EPEC strains was isolated by a hot phenol extraction protocol^86^. DNA in the RNA samples was digested using the TURBO DNase (Ambion), purified with phenol: chloroform: isopropanol and the quality was assessed using the Agilent RNA 6000 Nano Kit on the Agilent 2100 Bioanalyzer (Agilent Technologies). rRNA was depleted from total RNA samples using MICROBExpress (Ambion). Strand-specific RNA-seq cDNA library preparation of the total RNA of the different EPEC strains and barcode introduction was based on RNA adapter ligation as described previously^87^. Quality of the libraries was validated using Agilent 2100 Bioanalyzer (Agilent Technologies) following the manufacturer’s instruction. Cluster generation was performed using the Illumina cluster station. Single-end sequencing on the HiSeq2500 and Genome Analyzer IIx followed a standard protocol. The fluorescent images were processed to sequences and transformed to FastQ format using the Genome Analyzer Pipeline Analysis software 1.8.2 (Illumina). The sequence output was controlled for general quality features, sequencing adapter clipping and demultiplexing using the fastq-mcf and fastq-multxtool of ea-utils^88^.

### Read mapping, bioinformatics and statistics

Quality controlled/assessed libraries were mapped to the genome of *E.coli* strain E2348/69 (Acc.: chr: FM180568.1, plasmid pE2348-2: FM18070.1, plasmid pMAR2: FM180569.1) using *Bowtie2* (version 2.2.3)^89^ with default parametrization^90^. After read mapping, *SAMtools*^91^ was employed to filter the resulting BAM files for uniquely mapped reads (both strands) only. Reads were classified as uniquely mapped reads with a unique genomic location if and only if they could not be aligned to another location with a higher or same mapping quality. The resulting bam files constituted the basis for all further downstream analyses. For detailed mapping statistics, see Dataset S3. Reads aligned to annotated genes were quantified with the *htseq-count* program^14^ in *union* mode using the public NCBI gene annotations for *E. coli* strain E2348/69. The determined uniquely mapped read counts served as input to *DESeq2*^92^ for pairwise detection and quantification of differential gene expression. For *DESeq2* parametrization we used a beta prior and disabled the Cook distance cut off filtering. All other parameters remained unchanged. The list of *DESeq2* determined differentially expressed genes (DEGs) was filtered with a conservative absolute log_2_ fold change cutoff of at least 2, and a p-value cutoff corrected for multiple testing of at most 0.05. Lists of differentially expressed genes were further annotated with pathway information from the KEGG database. FASTQ files of all used libraries are deposited at the data repository GEO with the accession number GSE103415. Results of the comparative transcriptome analysis are given in Dataset S2.

### RNA CsrA electro mobility shift assay

CsrA binding to RNAs was determined by electro mobility shift assays (EMSA) using recombinant CsrA-His_6._ Template DNA for *in vitro* transcription was generated by PCR from E2348/69 EPEC genomic DNA, with primer sequences listed in the Supplementary Data Table S1. RNA was synthesized *in vitro* using the TranscriptionAid T7 High Yield Transcription Kit (Fermentas) and purified by phenol purification as described elsewhere. *In vitro* transcribed sRNA was biotin-labeled using 6 µl of 10x RNA-Ligase buffer (New England Biolabs (NEB)), 6 µl 10 mM ATP (NEB), 2 µg *wza* RNA, 6 µl DMSO (NEB), 6 µl 10 µM pCp-biotin (Jena Bioscience) and 18 µl 50% PEG-8000 (NEB) in a total volume of 60 µl at 17 °C overnight. RNA was incubated for 10 min at 70 °C before addition to the reaction mix. After biotinylation, RNA was purified via phenol-chloroform purification. In the binding reactions, 2 nM *hns* negative control RNA, 2 nM of *wza* 5’UTR RNA and 5 µl of different concentrations of CsrA-His_6_ were applied in a total volume of 10 µl. The reaction mix was incubated for 20 min on ice. *rovC* 5’ UTR was used as positive control with addition of 0 nM and 100 nM CsrA-His_6_. Reactions were separated on a native polyacrylamide gel using 1x TBE as electrophoresis buffer for 45–60 min at 80 V. Semidry transfer to a PVDF membrane (Thermofischer) was done at 20 V for 30 minutes, and RNA was crosslinked using UV light. Detection of biotinylated RNA was performed using the chemiluminescent nucleic acid detection module kit (Thermofischer). CsrA-His_6_ and control RNAs were purified as described in Heroven *et al*. 2008.

### Radiolabeling of RNA for CsrA electro mobility shift assays

For the labeling reaction, 200 ng of dephosphorylated RNA sample were incubated with 3 µl radioactive γ-P^32^-Adenosine 5’triphosphate (SRP-301, Hartmann Analytik), 1 µl polynucleotide kinase and 10x polynucleotide kinase buffer in a total volume of 10 µl for 1 h at 37 °C. Radiolabeled RNA was purified using the RNA Clean & Concentrator-5 kit (Zymo) according to the manufacturers’ protocol. Samples were eluted in 20 µl of RNase free water. Electro mobility shift assay was performed as described previously.

### Iodine staining of glycogen

Glycogen staining of the *ΔcsrA* mutant strain and wildtype strain was performed on Kornberg medium (1,1% K_2_HPO_4_, 0,85% KH_2_PO_4_, 0,6% yeast extract, 0,5% glucose, 1,5% agar) with staining the cells by iodine varpor as described by Bhatt *et al*.^23^. Additionally a separated staining was performed which was adapted from^93^. Staining was performed by carefully adding 5 ml of Lugol’s solution on top of the plate and incubation for 5 min at RT. After incubation, the Lugol’s solution was carefully removed.

## Supplementary information

Supplementary Information

S2

S3

S1
