# Supplementary material for: Metabolome and transcriptome-wide effects of the carbon storage regulator A in enteropathogenic Escherichia coli
Source: Sci Rep. 2019 Jan 15;9:138. doi: 10.1038/s41598-018-36932-w (PMC6333774; doi:10.1038/s41598-018-36932-w)
Supplement: Supplementary file 1 — Supplementary Information [file 41598_2018_36932_MOESM1_ESM.docx]

**Supplementary Files**

**Metabolome- and transcriptome-wide effects of the carbon storage regulator A in enteropathogenic *Escherichia coli*.**

Volker Berndt, Michael Beckstette, Marcel Volk, Petra Dersch and Mark Brönstrup

Supplementary Tables: 3

Table S1: Primer sequences for the construction of the EPEC E2348/69 ΔcsrA knockout mutant using the lambda red recombinase system. 3

Table S2: Bacterial strains and plasmids used in this study 3

Table S3: Detection of lyso-lipids. Lyso-lipid levels were compared between ΔcsrA knockout strain and the E23486/69 wildtype strain. 4

Supplementary Figures: 5

Figure S1: Growth curve of the ∆csrA deletion strain and the wildtype in either M9 (top) or MOPS (bottom) minimal media. 5

Figure S2: Iodine staining of the EPEC ∆csrA mutant and the wildtype 6

Figure S3: Volcano plot of all (assigned and unassigned) metabolome features 7

Figure S4: Volcano plot of all (assigned and unassigned) transcripts, filtered by significance 8

Figure S5: Gel mobility shift assay of the 5’-UTR of the wza RNA (colanic acid synthesis) 9

Figure S6: Uncropped images of western blot for all panels shown in Fig 1. 10

Figure S7: Influence of CsrA on metabolites of the citric acid cycle. 11

Figure S8: Gel mobility shift assay of the 5’-UTR of the cfa RNA encoding cyclopropane-fatty-acyl-phospholipid synthase. 12

Figure S9: Gel mobility shift assay of the 5’-UTR of the entC RNA encoding enterobactin biosynthesis 12

The following data sets can be downloaded as separate files: 13

Dataset S1.xls 13

Dataset S2.xls 13

Dataset S3.xls 13

# Supplementary Tables:

Table S1: Primer sequences for the construction of the EPEC E2348/69 Δ*csrA* knockout mutant using the lambda red recombinase system. The endogenous sequence of the *csrA* gene was replaced by the kanamycin cassette from the vector pKD4 (60). For further description see *Experimental Procedures*.

| primer | sequence |
| --- | --- |
| EPEC_csrA_F (upstream fragment) | TTAGCGGTGTTGAGCCGAAAATGTTGCGTACC |
| EPEC_csrA_R (downstream fragment) | TTC GTA GCC GAG TAC TCT ATC CAG CTG AGC TAC G |
| EPEC_5'_F | CTAAGGAGGATATTC ATATGCCAGGCTGAAAAATCCCAGCAGTCC |
| EPEC_5'_R | GAAGCAGCTCCAGCCTACACATCG ACG AGT CAG AAT C |
| pKD3-4_F | TGTGTAGGCTGGAGCTGCTTC |
| pKD3-4_R | CATATGAATATCCTCCTTAG |
| csra_F_PZ_nativ (csrA_forward+100) | TAGGTACCATTAACGCTATCGACAACG |
| EC_csrA_R (*csrA_*reverse) | TAGTCTAGATTA GTA ACT GGA CTG CTG G |
| fwd1_wza | GTAATACGACTCACTATAGTCTGGATGCCTGAAAGACC |
| rev_wza | ATCAATTTCATTTTGGATTTCATC |
| fwd1_wza | GTAATACGACTCACTATAGTCTGGATGCCTGAAAGACC |
| rev_wza2 | CATTGTTTATTTATCACTTTGGC |

# Table S2: Bacterial strains and plasmids used in this study

| Strains or plasmids | Relevant genotype | Reference or source |
| --- | --- | --- |
| EPEC 2348/69 | wild-type EPEC serotype O127:H6 | (80) |
| EPEC *ΔcsrA* | EPEC 2348/69 ∆*csrA* deletion | This study |
| EPEC *csrA plac::csrA* | EPEC 2348/69 transformed with vector pUC18_pLac::*csrA* | This study |
| EPEC TEM | E2348/69 *tir-bla* fusion | (67) |
| CC118 | *Δ(ara-leu)araDΔlacX74 galE galK phoA20 thi-1 rpsE rpoB argE(Am) recAl λpir* | (81) |
| pKD4 | empty vector, pANTSY backbone  FRT-kan-FRT, R6K | (60) |
| pUC18_pLac::*csrA* | *csrA* gene from EPEC 2348/69 under the control of the native and *lac* promoter cloned in the MCS of pUC18 vector | This study |

# Table S3: Detection of Lyso-lipids. Lyso-lipid levels were compared between Δ*csrA* knockout strain and the E23486/69 wildtype strain. Significant altered (p-value ≤ 0.05) metabolites are marked in red.

| Lipid | MS mode | Log2 fc | p-value |
| --- | --- | --- | --- |
| LysoPE(12:0) | Negative | -0.52 | 5.13E-03 |
| LysoPE(14:0) | Negative | -1.47 | 3-56E-06 |
| LysoPE(14:0) | Positive | -1.35 | 3.44E-03 |
| LysoPE(14:1) | Negative | -1.52 | 1.64E-05 |
| LysoPE(14:1) | Positive | -1.39 | 6.45E-04 |
| LysoPE(16:0) | Negative | -0.71 | 3.61E-02 |
| LysoPE(16:0) | Positive | -0.57 | 5.61E-01 |
| LysoPE(16:1) | Negative | -0.41 | 6.45E-02 |
| LysoPE(16:1) | Positive | -0.53 | 2.18E-01 |
| LysoPE(17:0)cyc | Negative | -1.95 | 7.77E-05 |
| LysoPE(17:0)cyc | Positive | -2.03 | 2.49E-03 |
| LysoPE(18:1) | Negative | -0.23 | 4.83E-01 |
| LysoPE(18:1) | Positive | -0.10 | 9.36E-01 |
| LysoPG(14:0) | Negative | -1.78 | 3.70E-05 |
| LysoPG(14:0) | Positive | -6.02 | 4.93E-03 |
| LysoPG(16:0) | Negative | -1.43 | 9.69E-05 |
| LysoPG(16:0) | Positive | -1.00 | 4.00E-01 |
| LysoPG(16:1) | Negative | -1.08 | 3.13E-04 |
| LysoPG(16:1) | Positive | -1.77 | 1.31E-03 |
| LysoPG(17:0)cyc | Negative | -3.61 | 1.07E-05 |
| LysoPG(17:0)cyc | Positive | -7.50 | 7.92E-04 |
| LysoPG(18:1) | Negative | -1.47 | 4.40E-04 |
| PE (10:0/16:1) | Negative | -2.91 | 4.36E-09 |
| PE (16:0/16:1) | Positive | -1.52 | 7.10E-03 |
| PE (16:0/17:1cyc) | Positive | 0.85 | 3.42E-01 |
| PE (16:1/14:0) | Positive | 1.20 | 4.45E-01 |
| PE (16:1/18:1) | Positive | -1.77 | 9.10E-03 |

# Supplementary Figures:


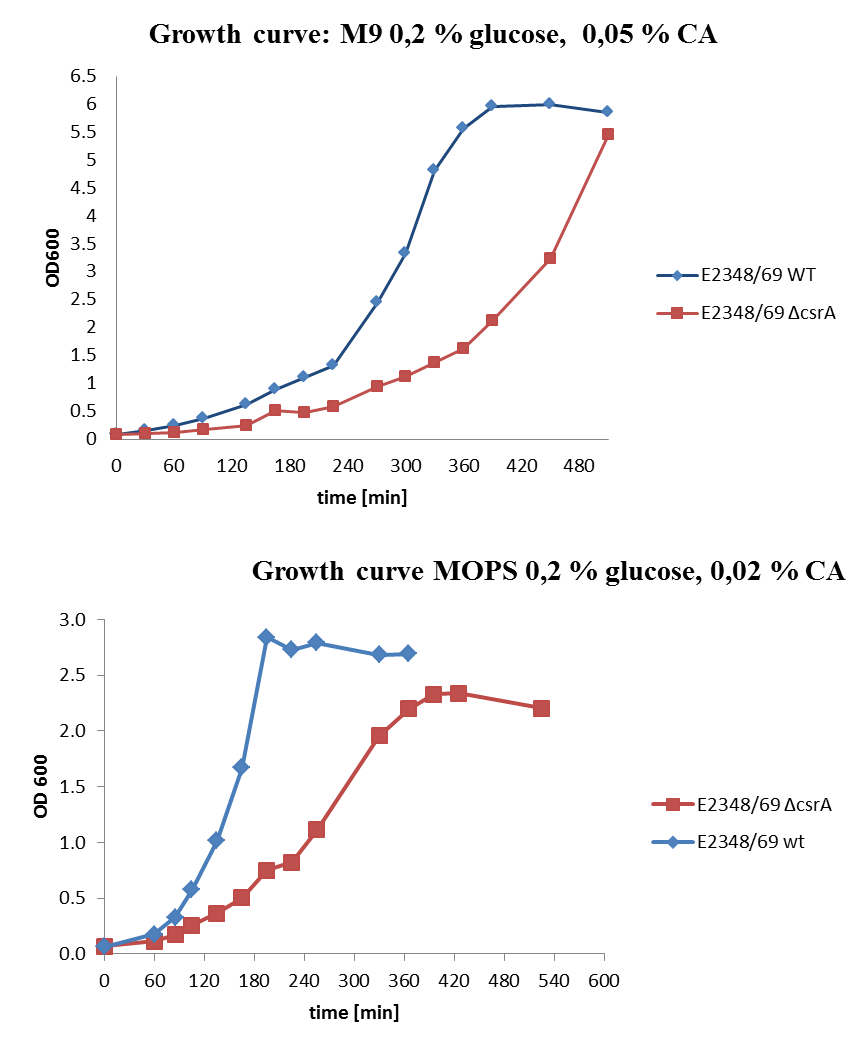


Figure S1: ***Growth curve of the* E2348/69 wildtype and the isogenic E2348/69 *∆csrA deletion strain in M9 (top) or MOPS (bottom) minimal media.*** *Media composition and growth conditions are described in Material and Methods.*


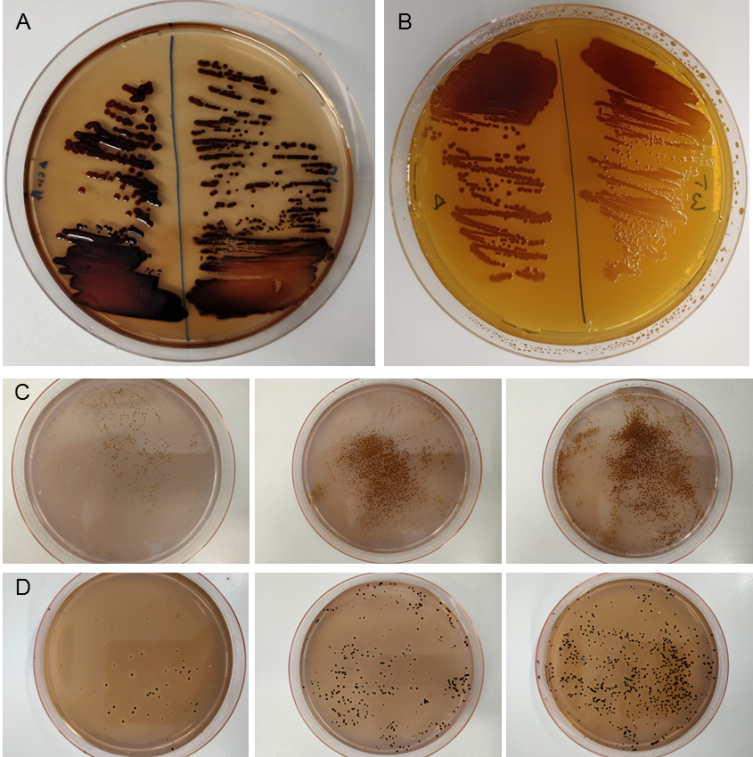


Figure S2: ***Iodine staining of the EPEC E2348/69 and its ∆csrA mutant derivative.*** *A: Staining with iodine vapour (left ∆csrA mutant, right wildtype), B: Staining with Lugol’s iodine solution (left ∆csrA mutant, right wildtype). C: Dilution of EPEC wildtype stained with iodine vapour, D: Dilution of ∆csrA mutant stained with iodine varpor. The ∆csrA mutant is slightly darker than the EPEC wildtype due to accumulation of glycogen. The morphology of the ∆csrA mutant is homogenous.*

C

**
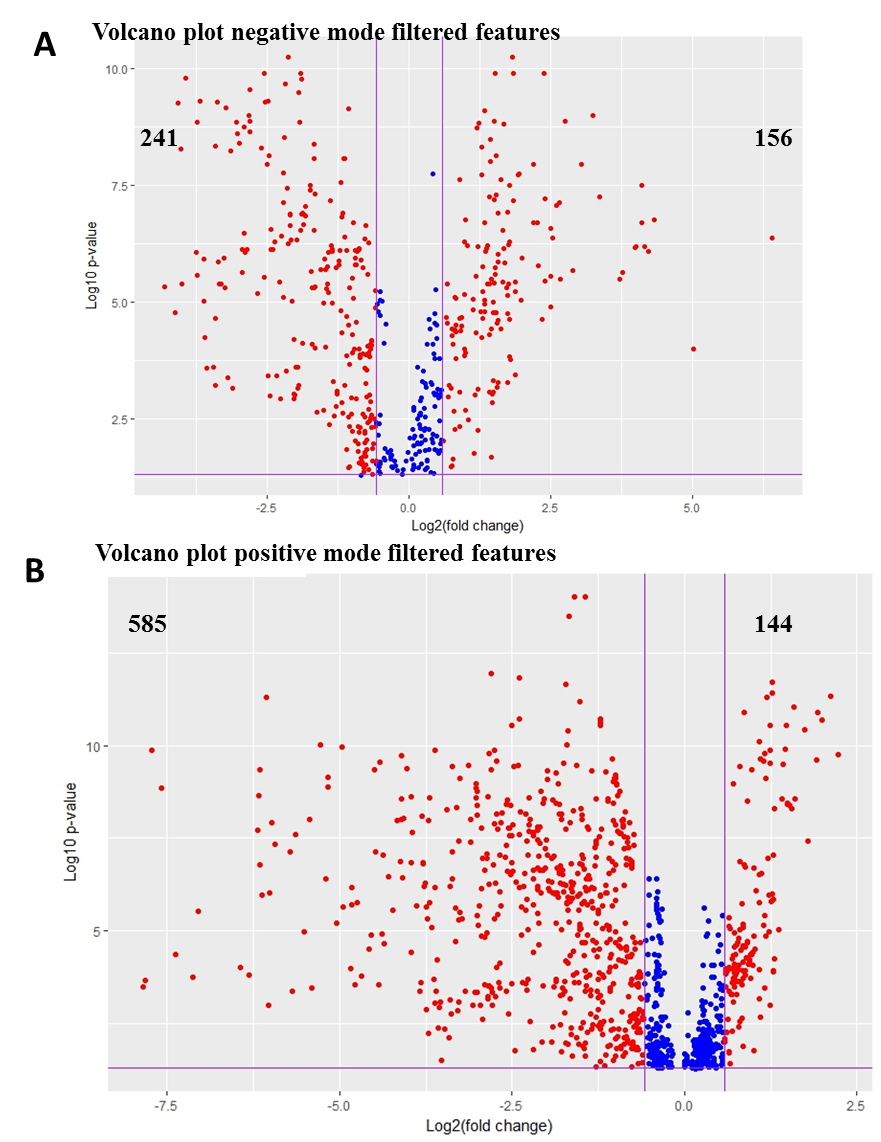
**

Figure S3: Volcano plot of all (assigned and unassigned) filtered metabolome features **between knockout and wildtype condition**. **A** *negative mode data: Of 515 total features, 156 were more abundant, and 241 were less abundant in the knockout strain.* ***B****: positive mode data: Of total 1056 features 144 were more abundant, and 585 were less abundant in the knockout strain. (Regulated features are defined by p-value ≥ 0.05, |fc| > 1.5. Please note, that the scaling of* *the x-axis is in log(2)fc.*


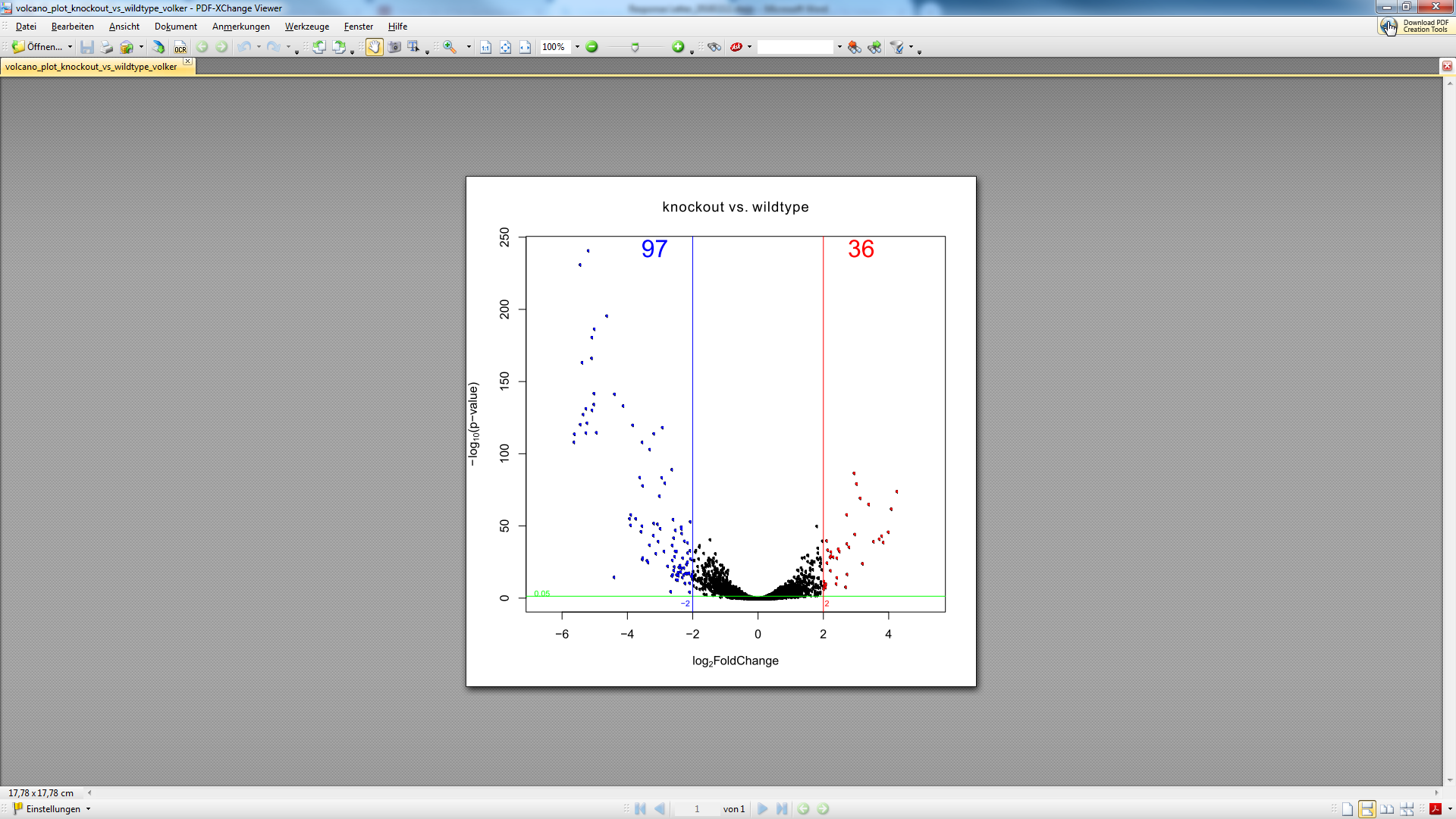


Figure S4: Volcano plot of all (assigned and unassigned) transcripts, filtered by significance (|logfc| ≥ 2.0 and a corrected p-value of ≤ 0.05). Of 4159 transcripts, 133 were significantly altered: 97 transcripts were less abundant in the knockout strain and 36 were more abundant.


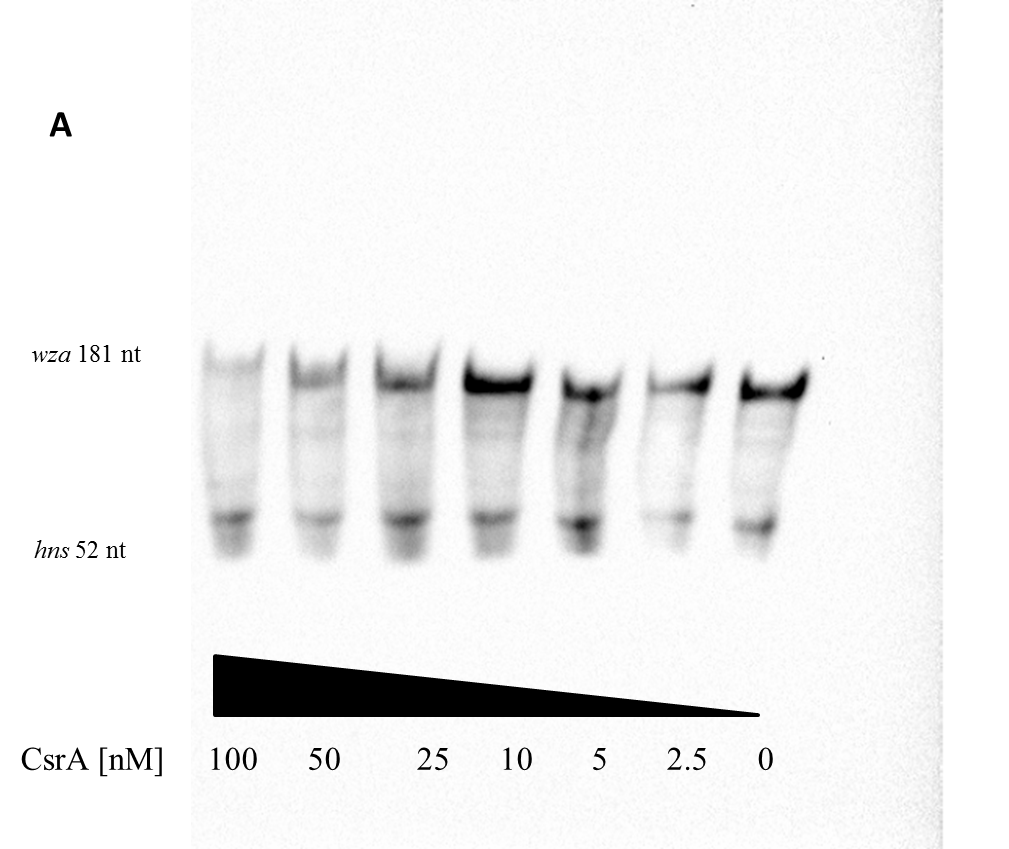


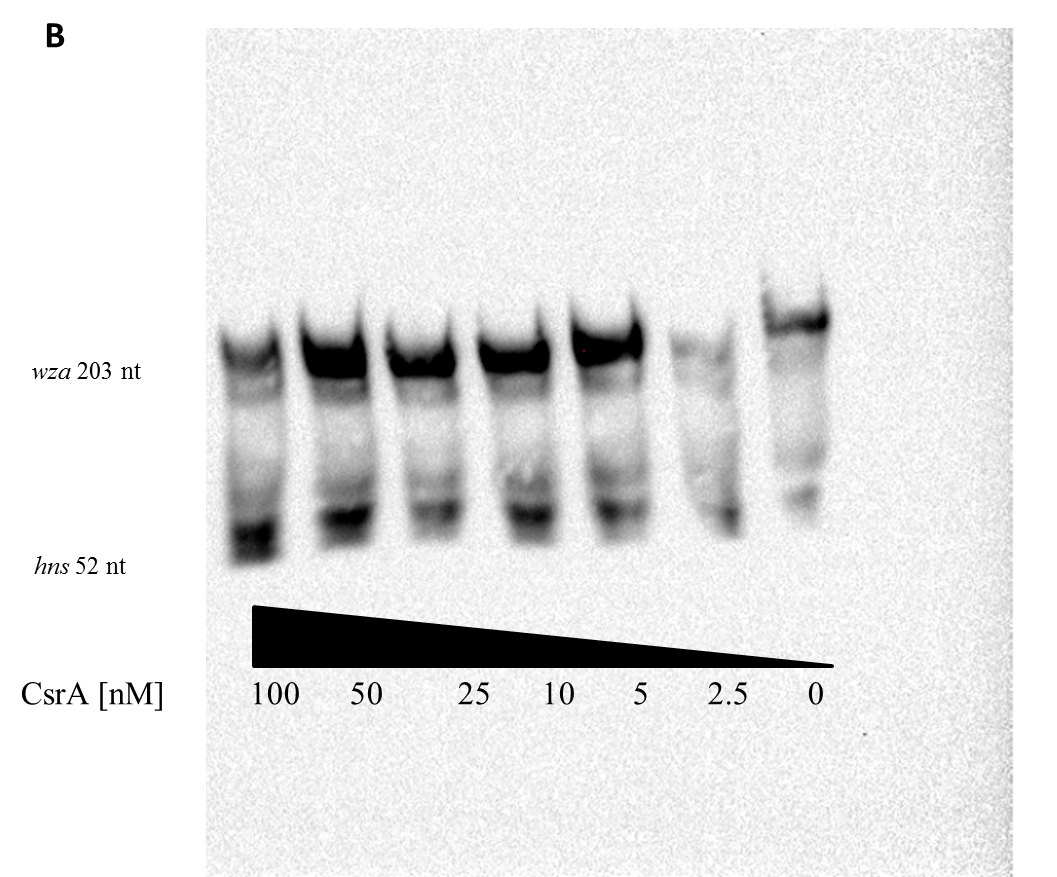


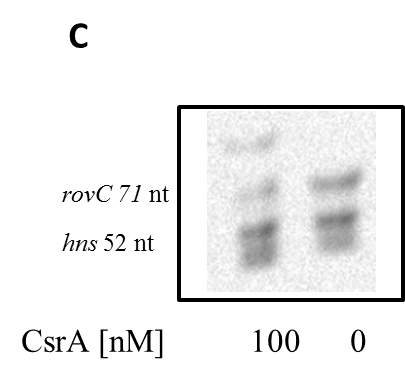


Figure S5: Gel mobility shift assay of the 5’-UTR of the wza RNA (colanic acid synthesis). *3'- biotinylated RNA of* wza *was incubated with increasing concentrations of CsrA. Gel shift assays were performed using biotinylated* hns *RNA as negative control. RovC-biotinylated* rovC *RNA was used as positive control at 0 nM and 100 nM CsrA concentration. No direct binding of CsrA to the* wza *5'-UTR fragment could be observed for the two tested RNA fragments (A.* wza*-UTR 181 nt, B.* wza*-UTR* *203 nt,* hns *52 nt,* rovC *77 nt)*

**
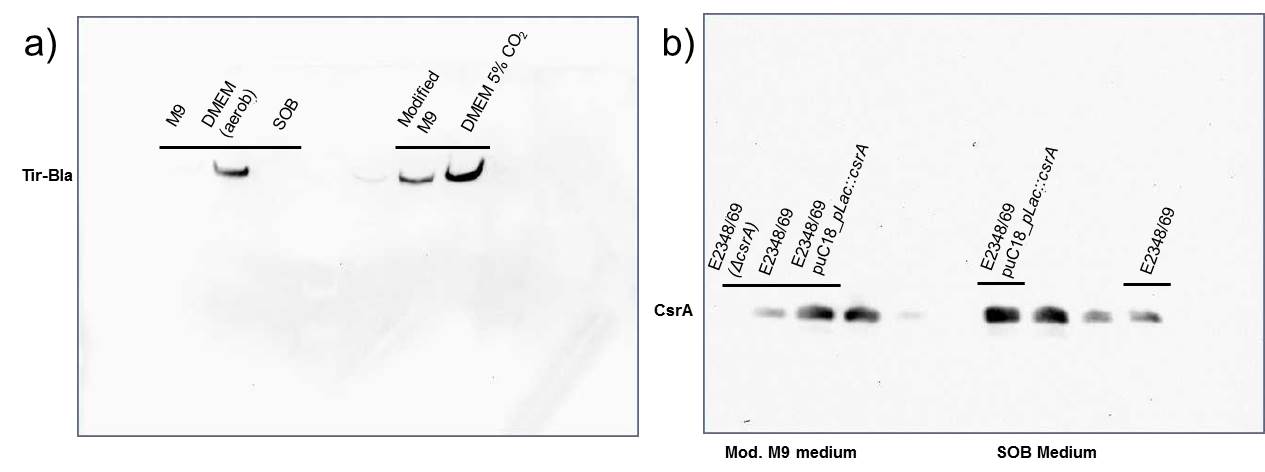
**

Figure S6: Uncropped images of western blot for all panels shown in Fig 1. *Unmarked bands are derived from different csrA overexpression vectors which were not further used for this study.*


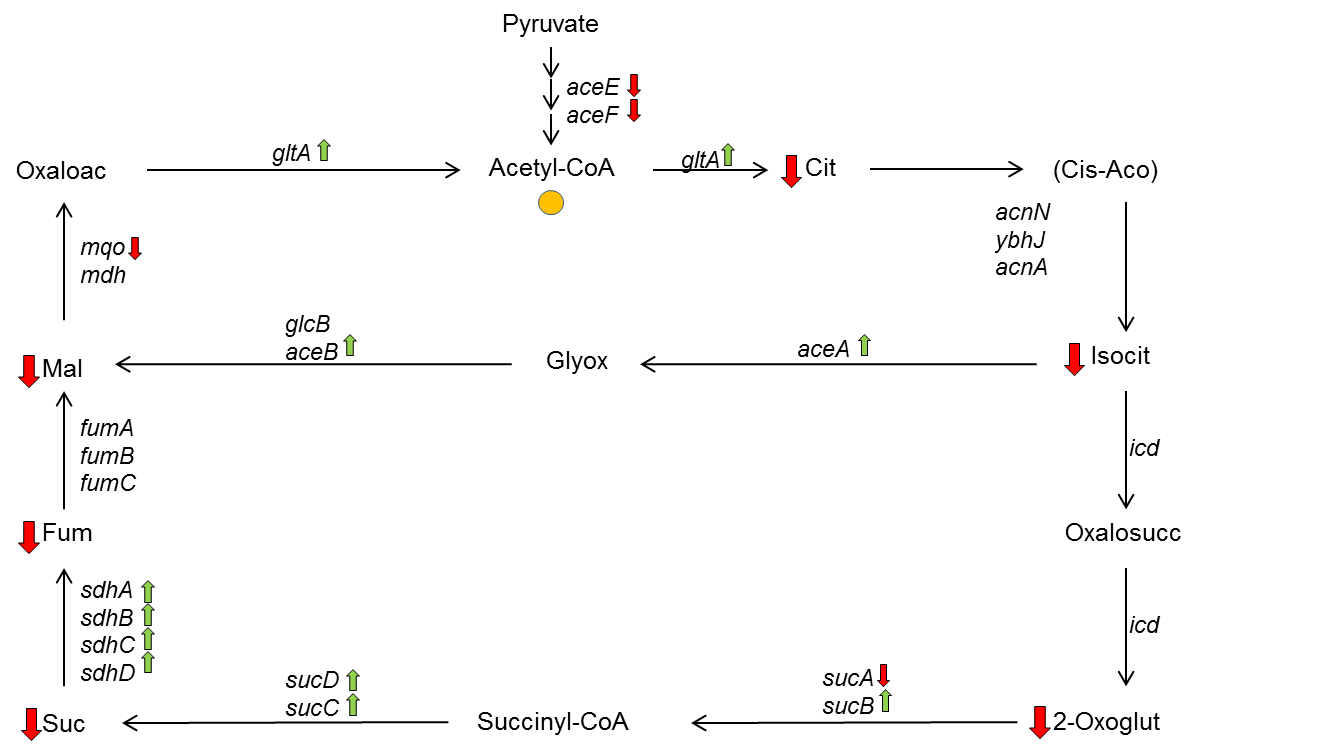


Figure S7: Influence of CsrA on metabolites of the citric acid cycle. Red arrows indicate downregulation, green arrows indicate upregulation, and yellow circles indicate unchanged levels in the *ΔcsrA* knockout strain compared to the wildtype. Metabolites: Cit citrate, Cis-Aco aconitic acid, Isocit isocitrate, Oxalosucc oxalosuccinate, 2-Oxoglut 2-oxoglutarate, Suc succinate, Fum fumarate, Mal malate, Oxaloac oxaloacetate, Glyox glyoxylate. For more details about the enzymes see Dataset S1.


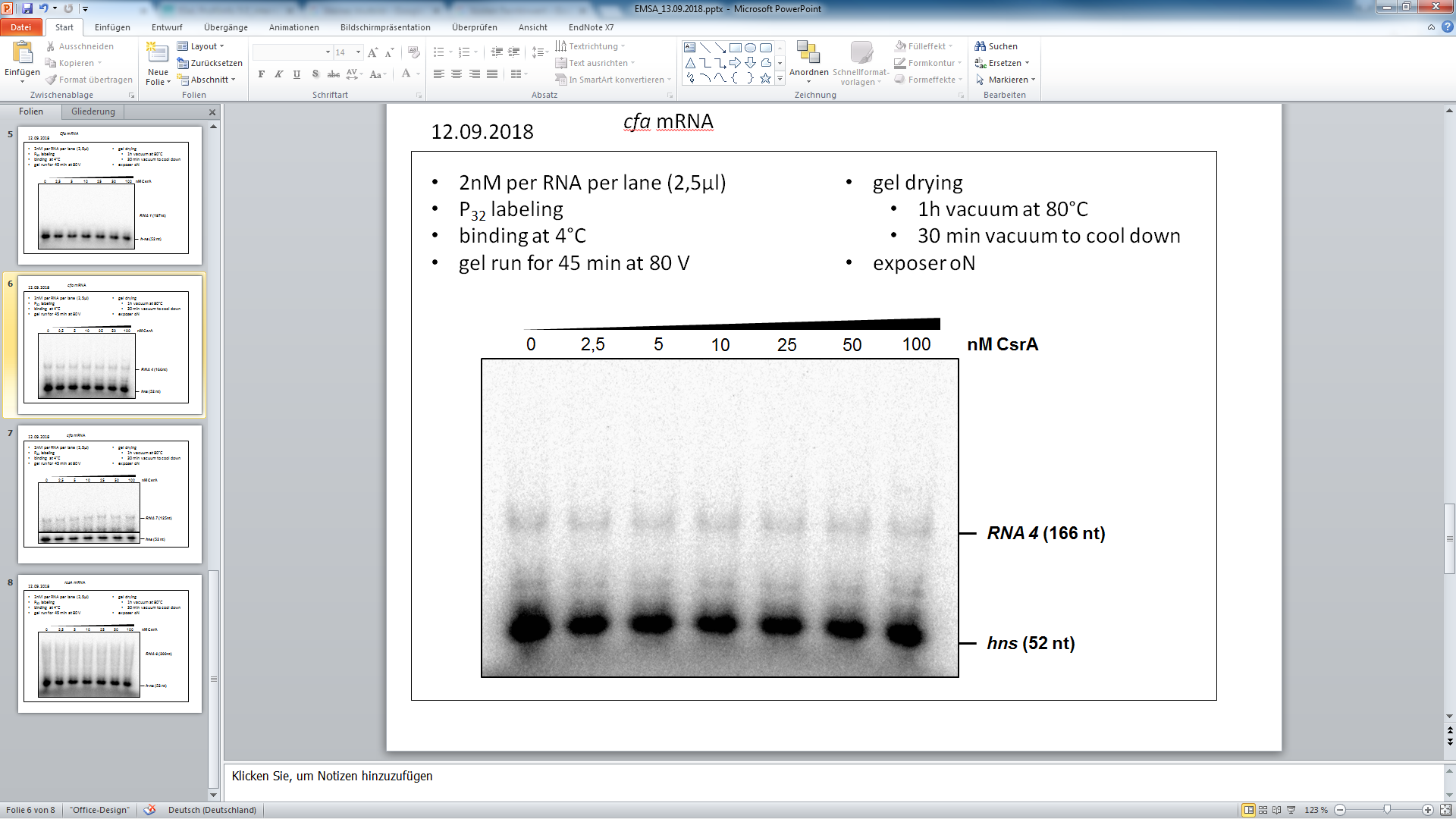


Figure S8: Gel mobility shift assay of the 5’-UTR of the cfa RNA encoding cyclopropane-fatty-acyl-phospholipid synthase. 3’ radiolabeled RNA of *cfa* was incubated with increasing concentrations of CsrA. *Electrophoretic mobility shift assays were performed using radiolabeled* hns *RNA as negative control (55 bp). A direct binding of CsrA to the cfa-UTR could not be observed for the tested RNA fragment (166 bp).*


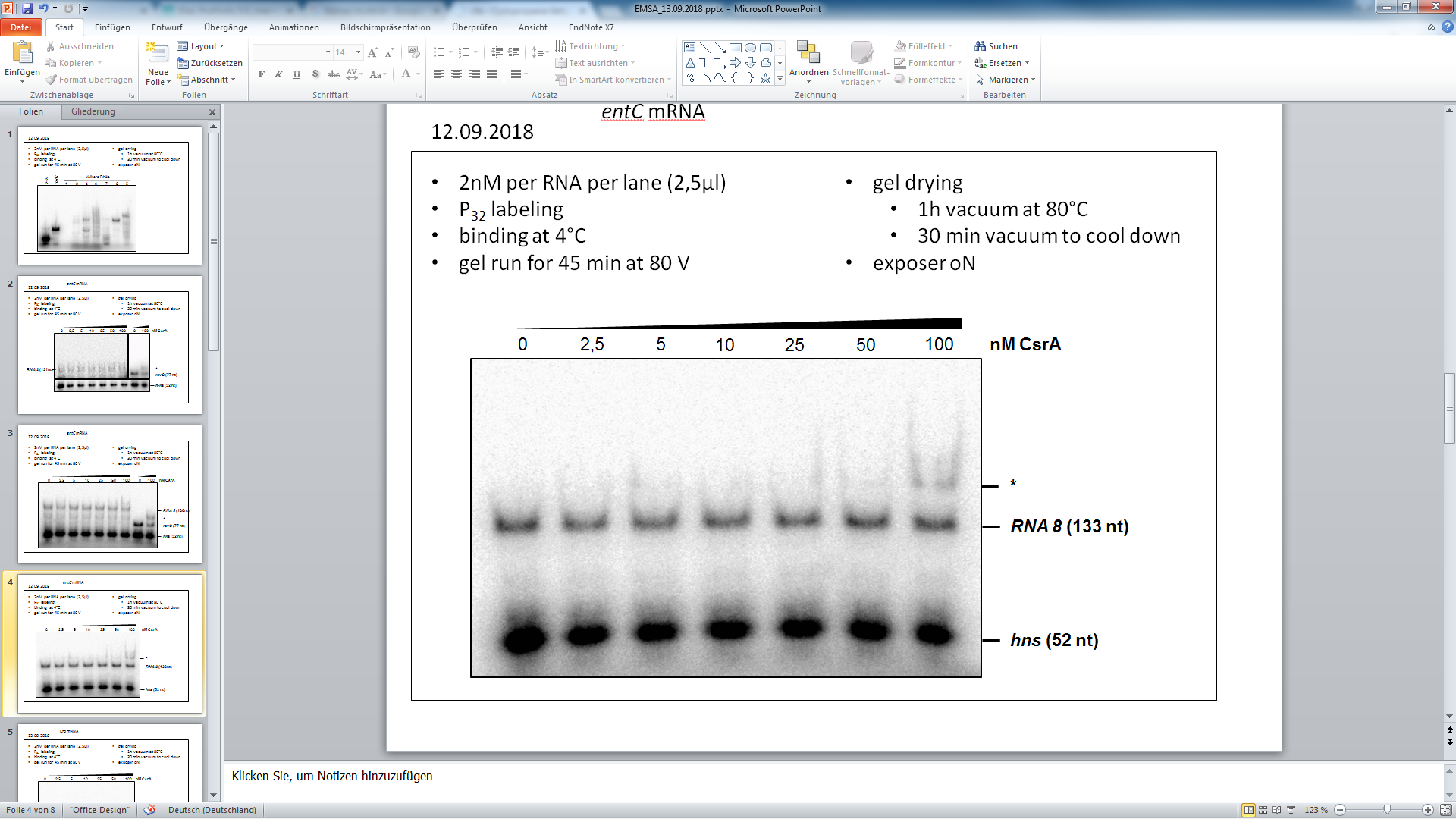


Figure S9: Gel mobility shift assay of the 5’-UTR of the entC RNA encoding enterobactin biosynthesis. 3’ radiolabeled RNA of *ent*C was incubated with increasing concentrations of CsrA. Electrophoretic mobility shift assays were performed using radiolabeled *hns* RNA as negative control (55 nt). * indicates *entC* RNA-CsrA complex formation (RNA 8, 133 nt).

# The following data sets can be downloaded as separate files:

# Dataset S1.xls

# Dataset S2.xls

# Dataset S3.xls
